# A Bayesian inference transcription factor activity model for the analysis of single-cell transcriptomes

**DOI:** 10.1101/gr.265595.120

**Published:** 2021-07

**Authors:** Shang Gao, Yang Dai, Jalees Rehman

**Affiliations:** 1 Department of Bioengineering, University of Illinois at Chicago, Chicago, Illinois 60612, USA;; 2 Department of Medicine, Division of Cardiology, University of Illinois at Chicago, Chicago, Illinois 60612, USA;; 3 Department of Pharmacology and Regenerative Medicine, University of Illinois at Chicago, Chicago, Illinois 60612, USA;; 4 University of Illinois Cancer Center, Chicago, Illinois 60612, USA

## Abstract

Single-cell RNA sequencing (scRNA-seq) has emerged as a powerful experimental approach to study cellular heterogeneity. One of the challenges in scRNA-seq data analysis is integrating different types of biological data to consistently recognize discrete biological functions and regulatory mechanisms of cells, such as transcription factor activities and gene regulatory networks in distinct cell populations. We have developed an approach to infer transcription factor activities from scRNA-seq data that leverages existing biological data on transcription factor binding sites. The Bayesian inference transcription factor activity model (BITFAM) integrates ChIP-seq transcription factor binding information into scRNA-seq data analysis. We show that the inferred transcription factor activities for key cell types identify regulatory transcription factors that are known to mechanistically control cell function and cell fate. The BITFAM approach not only identifies biologically meaningful transcription factor activities, but also provides valuable insights into underlying transcription factor regulatory mechanisms.

Single-cell RNA sequencing (scRNA-seq) is a powerful experimental technique to investigate the transcriptomic heterogeneity of individual cells within a tissue and uncover novel subpopulations of cells with distinct biological functions. It has successfully identified subtypes of mature differentiated cells in distinct tissues (*Tabula Muris* et al. 2018) as well as distinct developmental stages of progenitor subpopulations and the underlying cues regulating cell fate decisions (Shapiro et al. 2013; Zeisel et al. 2015; Regev et al. 2017; Zheng et al. 2017; Svensson et al. 2018). The characterization of subpopulations within a given tissue is commonly performed by examining the cell-to-cell similarities on their gene expression profiles. There has been a rapid development of methods and tools for scRNA-seq data analysis (Zappia et al. 2018). Most analytical methods use a form of gene expression data transformation to produce lower dimensional representations of scRNA-seq data to better capture the distances between cells (Amir et al. 2013; Satija et al. 2015; Žurauskienė and Yau 2016; Duren et al. 2018; Lopez et al. 2018; Wang and Gu 2018; Wu et al. 2018; Jung et al. 2019; Linderman et al. 2019; Moon et al. 2019). However, the identification of cell clusters based on the proximity of individual cells in lower dimensional space does not take into account the biological context (Kiselev et al. 2019). Therefore, downstream analyses based on these representations do not necessarily identify subpopulations of cells with defined biological functions. In addition, these methods do not provide an immediate means to uncover regulatory mechanisms in the identified subpopulation of cells.

We surmised that integrating the vast amount of known biological data on transcription factor binding sites could be leveraged to analyze scRNA-seq data. We therefore introduced a Bayesian hierarchical model that uses existing transcription factor ChIP-seq data for the inference of transcription factor activities in scRNA-seq data, which in turn can be used for downstream analysis such as identifying cell clusters based on distinct inferred transcription factor activities as well as generating a weighted hierarchy of target genes.

## Results

### Overview of BITFAM

**Figure 1.**
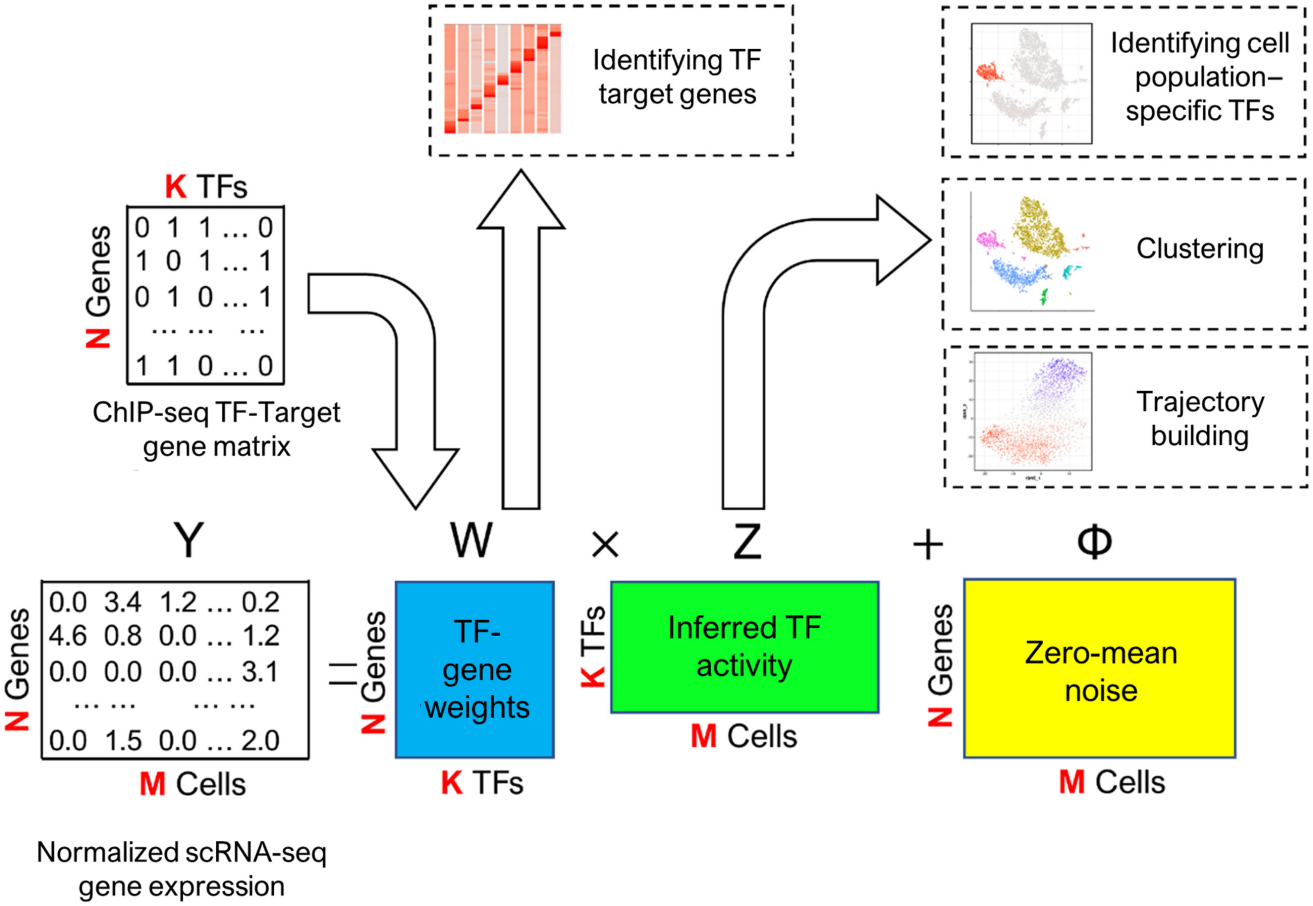
Overview of the BITFAM model. The input to BITFAM is the log-normalized scRNA-seq data and a binary matrix with the predicted target genes for each transcription factor obtained from ChIP-seq data. A Bayesian factor analysis model with regulatory prior knowledge is built to learn matrices of transcription factor activities and transcription factor targets. The log-normalized scRNA-seq data, matrix *Y*, are decomposed to matrix *W* and matrix *Z*. The ChIP-seq data are incorporated as different prior distributions in matrix *W*. The posterior distributions of matrix *W* and matrix *Z* are inferred by the variational method (Ghahramani and Matthew 2000; Wainwright and Jordan 2008). The final matrices of *W* and *Z* used for downstream analysis are constructed by taking the means of 300 random samples from the posterior distributions. The matrix *W* is used to identify the target genes of transcription factors in the specific data set. The matrix *Z* is used in clustering and trajectory analysis as well as providing insights into the transcription factor regulatory activities.

Our Bayesian inference transcription factor activity model (BITFAM) is based on a fundamental biological principle that the differences in scRNA-seq profiles of individual cells reflect distinct underlying transcription factor activity states (Fig. 1). Specifically, we assemble a set of factors in the model by associating each of them with a transcription factor's predicted target gene set that is obtained from GTRD databases of ChIP-seq data that has more than 17,485 transcription factor ChIP-seq samples (Yevshin et al. 2019). This information of known ChIP-seq data is used as a prior probability to guide the factorization of scRNA-seq data in a Bayesian factor analysis (BFA) model (Bai and Li 2012). BFA is an inference model that has been applied to capture heterogeneity in gene expression by considering generic pathways and gene sets as factors (Leek and Storey 2007; Hand 2013; Buettner et al. 2017). The model seeks to decompose an observed log-transformed normalized gene expression profiles (matrix *Y*) into a product of two matrices, *W* and *Z*. The rows of *Y* correspond to genes (N) and columns correspond to cells (M). The matrix *Z* is a scheme to generate alternative profiles of single-cell data. The matrix *W* represents factor loadings. Φ is the unobserved stochastic noise term with zero mean and a finite variance.

To guide the construction of biologically meaningful matrices *W* and *Z*, we embedded a structure in *W* by using the consensus transcription factor ChIP-seq data. Specifically, each column of *W* represents a transcription factor, and each row represents its potential targets, which are determined by the binding sites of the transcription factors in the ChIP-seq data. Because the existing ChIP-seq data are not specific to the cellar context where the single-cell profiles of interest are generated, we incorporated this information as prior probabilities on the elements in *W* and let the Bayesian inference procedure learn the posterior distributions of *W* and *Z* from the observed single-cell profiles. By doing so, the inferred matrix *W* determines the transcription factor targets according to the data of context.

A column of the matrix *Z* can be interpreted as the inferred transcription factor regulatory activities in the corresponding cell. As the number of transcription factors is much smaller than the number of the genes profiled, this model achieves dimension reduction of single-cell RNA-seq data. The matrix *W* can be interpreted as the regulatory weights between the transcription factors and genes. The values in a column of matrix *W* can be used to determine the most probable target genes of one transcription factor in the specific data set, a valuable insight because aggregate information on all potential transcription factor target genes does not provide a ranking of target genes. The matrices *Z* and *W* could be applied to several downstream analyses, including (1) the decomposition of the single-cell transcriptomic profile into transcription factor activities, (2) identifying a ranking of transcription factor target genes for each scRNA-seq data set, and (3) performing downstream analyses, such as clustering of cell subpopulations.

We next applied our model to the following scRNA-seq data sets: the *Tabula Muris* data sets that provide scRNA-seq data on all major organs of the mouse during adult homeostasis (*Tabula Muris* et al. 2018), a blood cell development data set that contains two differentiation trajectories from common myeloid progenitors (CMP) toward megakaryocyte–erythroid progenitors (MEP) and granulocyte-macrophage progenitors (GMP) (Paul et al. 2016), and a CRISPRi scRNA-seq data with 50 targeted CRISPR-deletions of transcription factors (Genga et al. 2019). Descriptions of all the analyzed data sets are provided in the Supplemental File (Supplemental Fig. S1). Cells in the first two data sets have been experimentally labeled by cell type using either antibodies or gene expression profiles and therefore can be used for evaluating the accuracy and functional relevance of BITFAM for assessing distinct cell population phenotypes. The CRISPRi data set can be used as a biological validation or ground truth of our inferred transcription factor activities because CRISPR-targeted deletion or knockdown of a transcription factor should reduce the corresponding transcription factor activity, even though the transcription factor activity will not be reduced to zero because CRISPR does not have complete deletion efficiency. The transcription factor ChIP-seq data were chosen from GTRD, a comprehensive database of transcription factor binding sites (TFBSs) identified from ChIP-seq experiments for human and mouse (http://gtrd.biouml.org) (Yevshin et al. 2019).

### Transcription factor activities inferred by BITFAM correspond to known biological functions

We investigated whether the transcription factor (TF) activities inferred by BITFAM for each cell are biologically meaningful. We show the results in two data sets as examples: The *Tabula Muris* lung data set (*Tabula Muris* et al. 2018) and the blood cell development data set (Paul et al. 2016). They represent two typical scenarios of experimental studies in discrete and continuous biological situations.

**Figure 2.**
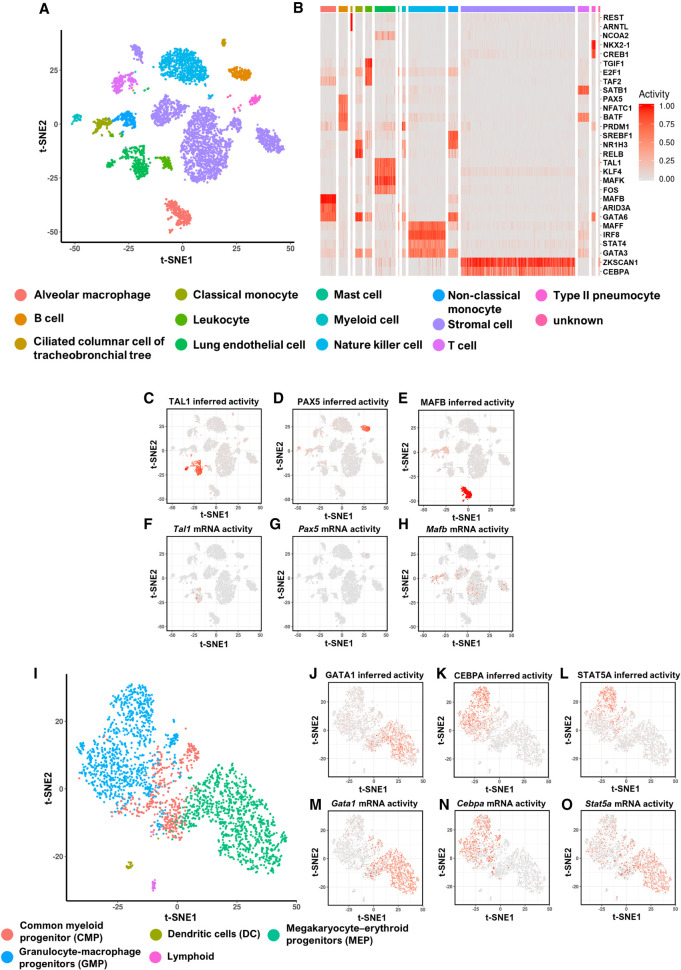
Transcription factor activities inferred by BITFAM correspond to known biological functions. (*A*) t-SNE plot of the *Tabula Muris* lung data set in which cells are colored by biologically defined cell types. The input to t-SNE algorithm is the log-normalized scRNA-seq profile. (*B*) Heatmap of inferred activities of specific transcription factors for each cell type in the lung. The columns are the cells and are grouped by cell type. The rows are the inferred transcription factor activities. (*C*–*E*) Inferred activities of TAL1, PAX5, and MAFB in the *Tabula Muris* lung data, respectively. (*F*–*H*) Log-normalized mRNA expression of *Tal1*, *Pax5*, and *Mafb* in the *Tabula Muris* lung data. (*I*) t-SNE plot of blood cell development data colored with cell types. (*J*–*L*) Inferred activities of GATA1, CEBPA, and STAT5A in the blood cell development data set. (*M*–*O*) Log-normalized mRNA expression levels of *Gata1*, *Cebpa*, and *Stat5a* in the blood cell development data set.

The *Tabula Muris* lung data set has 16 distinct cell types including epithelial cells, endothelial cells, lymphocytes, and macrophages visualized in a t-SNE plot with gene expression profiles (Fig. 2A). The cell types of the distinct subpopulations are labeled based on the expression of cell type–specific marker genes. The rules of selecting TFs when applying BITFAM are (1) the transcription factor is among the most variably expressed genes, (2) there is ChIP-seq data available for the TF, and (3) the transcription factor has at least 10 target genes among the most variably expressed genes. Using these criteria for the *Tabula Muris* lung data set, BITFAM inferred the activities of 106 transcription factors (Supplemental Fig. S2). These default criteria for BITFAM can be modified to include any transcription factor for which ChIP-seq data is available and users can thus easily add additional transcription factors that they are interested in into the learning list even if these additional transcription factors are not among the most variably expressed. The number of transcription factors and the redundancy of transcription factors did not significantly impact the inferred transcription factor activities or downstream analyses such as visualization of clusters, thus suggesting robustness of the model in the setting of multiple transcription factors that may have significant proportions of overlapping target genes (Supplemental Figs. S3, S4).

One of the utilities of inferring TF activities for each cell is the potential to generate profiles of cell type–specific TF activities. For this purpose, we used a random forest model to identify the inferred TF activities that were associated with specific cell types. For example, we labeled cells of a given cell type (identified by a biological label such as marker genes) as 1 and all other cells as 0. The random forest model is built using this cell label and the inferred transcription factor activities from the BITFAM *Z* matrix. By applying the same model to each cell type, we generated a landscape of inferred TF activities across all cell types. The heatmap of the inferred TF activities in the *Tabula Muris* lung data set (Fig. 2B) showed a distinctive pattern of TF activation in biologically defined cell types. We found that TAL1 had a high inferred transcription factor activity in endothelial cells, with an inferred activity in 99% (using the 75th percentile of the inferred transcription factor activities as a cutoff) of lung endothelial cells (Fig. 2C). PAX5 and EBF1 were the transcription factors with the highest inferred activities in B cells. MAFB was the transcription factor with the highest inferred activity in alveolar macrophages (Fig. 2D,E; Supplemental Fig. S5). However, *Tal1* mRNA in the scRNA-seq data was only detected in 25% of endothelial cells (Fig. 2F), and the mRNA of *Pax5* and *Mafb* was even lower in the B cells and macrophages (Fig. 2G,H), thus suggesting that inferring the transcription factor activities via BITFAM complements approaches that focus on analyzing mRNA levels of TFs. The area under receiver operating characteristic curve (AUROC) is used to show how specific inferred transcription factor activity is for a cell type that is independently defined using the established cell identify markers (already provided by the *Tabula Muris* Consortium). For example, BITFAM inferred PAX5 activity for each cell in the data set. For this analysis, the ground truth was the identity of the cells as categorized by the *Tabula Muris* Consortium using cell-specific markers. B cells were assigned to the positive class, whereas all other cells were assigned to the negative class. We then associated the PAX5 inferred activities with the positive and negative classes, thus allowing us to generate an AUROC for each inferred transcription factor activity in each cell population. We found that the inferred activities for TAL1, PAX5, and MAFB had markedly higher AUROC values than those which would have solely relied on measures transcription factor mRNA levels (Supplemental Fig. S6A–C). By comparing t-SNE visualization of the BITFAM-inferred transcription factor activities with those of transcription factor mRNA expression (Supplemental Fig. S7), we observed that inferred transcription factor activities can segregate biologically defined cell subsets, whereas mRNA levels do not.

For the blood cell development data set (Fig. 2I), GATA1-inferred activity was the highest in megakaryocyte–erythroid progenitors (MEPs) (Fig. 2J), whereas the inferred activities of CEBPA and STAT5A were the highest in granulocyte-macrophage progenitors (GMPs) (Fig. 2K,L). The level of *Gata1* mRNA expression (Fig. 2M) and *Cebpa* mRNA expression (Fig. 2N) corresponded to the inferred activities, but the mRNA levels of *Stat5a* were not restricted to GMPs (Fig. 2O). Although the AUROC of the inferred GATA1 activities were similar to those of the *Gata1* mRNA as a marker of MEPs (Supplemental Fig. S6D), the AUROC values for the inferred activities were substantially higher for CEBPA and STAT5A (Supplemental Fig. S6E,F). These results indicate that the inferred activities derived from BITFAM do not necessarily correspond to the mRNA expression levels of the transcription factors. This is especially important because limited sequencing coverage in scRNA-seq may inadequately capture the mRNA levels of key regulatory genes in individual cells.

### BITFAM generates a ranking of preferred transcription factor target genes using scRNA-seq data

**Figure 3.**
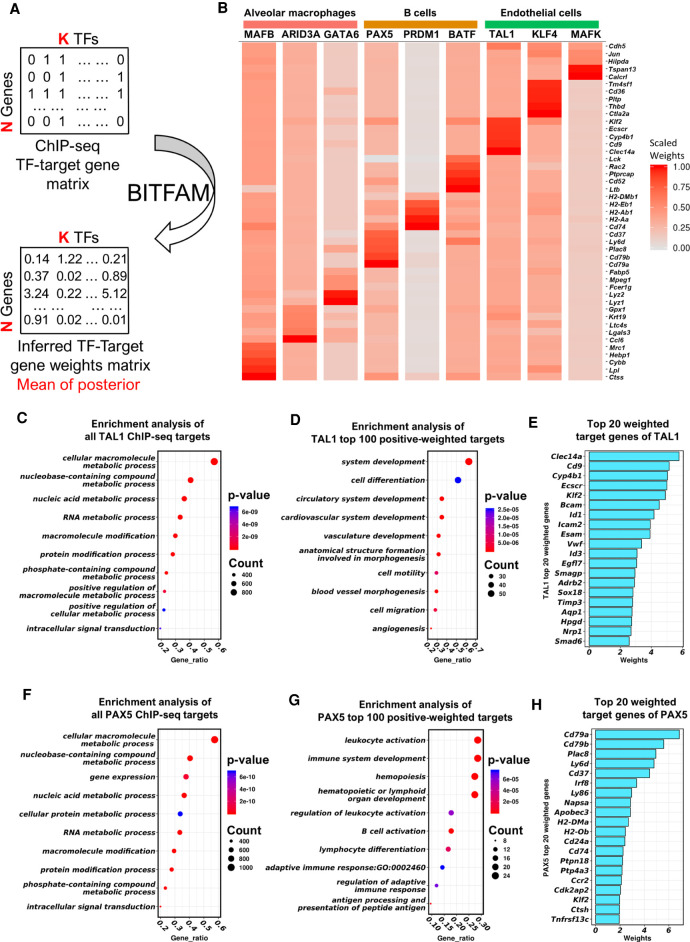
BITFAM generates a ranking of preferred transcription factor target genes using scRNA-seq data. (*A*) The weights between the transcription factors and target genes inferred by BITFAM are based on ChIP-seq data and scRNA-seq data and identify preferred target genes in the specific data set. (*B*) Heatmap of five top-weighted target genes of transcription factors learned from *Tabula Muris* lung data. For each cell type, we depict TFs that were identified by BITFAM to be highly active in a given cell type. We list the top-weighted target genes based on the weights in the *W* matrix learned from the model and which are rescaled to [0,1] within each TF to generate the heatmap. (*C*) The top 10 significant GO terms of TAL1 ChIP-seq target genes in the *Tabula Muris* lung data. (*D*) The top 10 significant GO terms of TAL1 top 100 positive weighted genes learned from the *Tabula Muris* lung data. (*E*) The top 20 weighted target genes of TAL1. (*F*) The top 10 significant GO terms of PAX5 ChIP-seq target genes in the *Tabula Muris* lung data. (*G*) The top 10 significant GO terms of PAX5 top 100 positive weighted genes learned from the *Tabula Muris* lung data. (*H*) The top 20 weighted target genes of PAX5.

We further examined the biological significance of the learned weight matrix *W* in the BITFAM model. The GTRD database integrates the ChIP-seq transcription factor binding data obtained from distinct cell types and biological conditions. In such a comprehensive transcription factor target gene list, many transcription factors have thousands of potential target genes; however, it is very likely that in any given cell type only a small fraction of these potential target genes is truly being targeted by a transcription factor. BITFAM learns the weights for potential transcription factor target gene pairs in every data set. This allows BITFAM to generate a ranking of target genes for any given transcription factor based on the mean of the posterior distribution of the weights (Fig. 3A).

We selected the transcription factors that were inferred to be specifically activated in alveolar macrophages, B cells, and endothelial cells, and examined the top-weighted target genes learned by BITFAM in the *Tabula Muris* lung data set (Fig. 3B). We performed a Gene Ontology (GO) enrichment analysis on the top 100 genes with the highest positive weights and compared this to the GO analysis on all variably expressed ChIP-seq target genes. For example, the overall TAL1 ChIP-seq target genes were enriched for generic biological processes such as nucleic acid metabolism and protein modification (Fig. 3C). On the other hand, the TAL1 top-weighted target genes identified by BITFAM, were enriched for cell type–specific processes such as vasculature development and angiogenesis (Fig. 3D). The top 20 weighted genes of TAL1 (Fig. 3E) include genes such as *Clec14a*, which is a recently identified key regulator of blood vessel development and vascular function (Lee et al. 2017), consistent with the biologically established role of TAL1 in vascular development (Lazrak et al. 2004). The transcription factor PAX5, on the other hand, is known to be highly active in B cells (Nutt et al. 1999), but this was not readily determined when analyzing all PAX5 ChIP-seq target genes, which were again enriched for general biological processes such as nucleic acid metabolism, macromolecule modification, and protein modification (Fig. 3F). However, when we analyzed the top PAX5 learned target genes using BITFAM, we found that these target genes were enriched for B cell activation and B cell–associated immune responses (Fig. 3G), thus providing important insights into the biological function of PAX5, which had been independently and experimentally determined by *Pax5* deletion studies (Liu et al. 2014). The top 20 weighted genes of PAX5 shown in Figure 3H again highlight key genes involved in B cell identity. We also conducted the same enrichment analysis on randomly sampled 100 ChIP-seq target genes or the most expressed ChIP-seq target genes. The enrichment analysis identified pathways involved in general cellular function but did not identify B cell–specific pathways, thus suggesting that the BITFAM learned preferred target genes may provide more functional insights about a cell population's function than a mere random choice of possible ChIP-seq target genes (Supplemental Fig. S8). We next investigated whether the negatively weighted genes identified by BITFAM were indeed ChIP-seq target genes and what their functional relevance could be for PAX5 and MAFB as illustrative examples. As shown in Supplemental Figure S9, the negatively weighted genes for the B cell development TF PAX5 are enriched in genes from the GO term related to T cell activation, consistent with the notion that PAX5 could suppress T cell activation and differentiation while promoting B cell development. Genes with the most negative weights for PAX5 and MAFB were indeed ChIP-seq target genes.

These results showed BITFAM's ability to learn target genes of the transcription factors specific to their biological function and would allow for the inference of preferred target genes and functions of transcription factors.

### The learned transcription factor activity profiles can be used for downstream analysis

**Figure 4.**
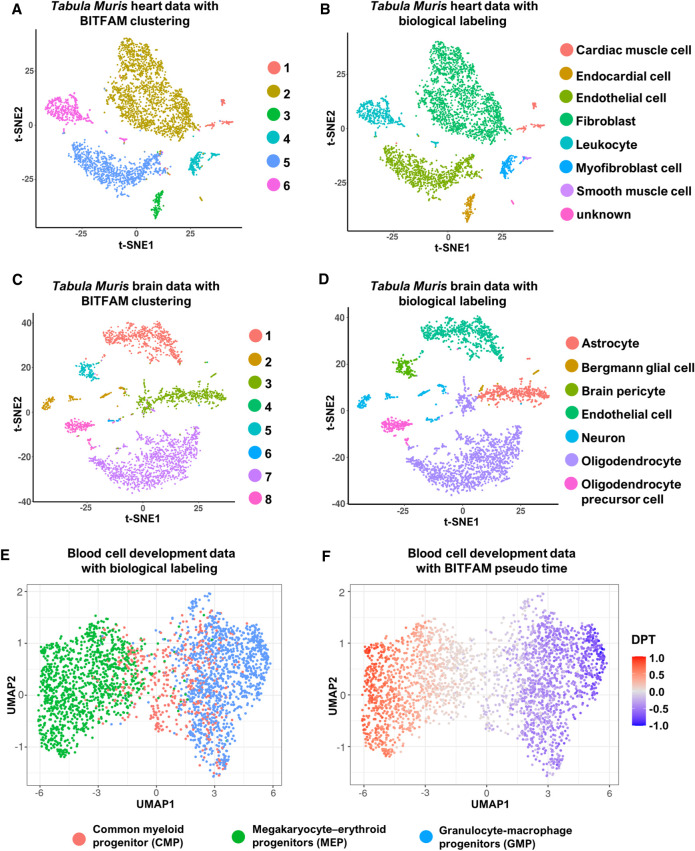
Clustering of cell subpopulations by inferred transcription factor activities. (*A*) t-SNE plot of the *Tabula Muris* heart data set in which cells are colored by BITFAM clusters. (*B*) t-SNE plot of the *Tabula Muris* heart data set in which cells are colored by biologically defined cell types. (*C*) t-SNE plot of the *Tabula Muris* brain data set in which cells are colored by BITFAM clusters. (*D*) t-SNE plot of the *Tabula Muris* brain data set in which cells are colored by biologically defined cell types. (*E*) UMAP plot of the inferred transcription factor activities in the blood cell development data set with cells colored by biologically defined cell types. (*F*) UMAP plot of the inferred transcription factor activities colored by the pseudo-time values calculated by the standard DPT workflow.

We next explored how inferred transcription factor activities could be used for downstream analyses and whether activity patterns would reflect distinct functions of cell subpopulations. We applied Louvain's algorithm on the inferred transcription factor activities (Methods) to assess cell–cell inferred transcription factor activity distances and identify cell clusters, which were visualized by t-SNE. In the *Tabula Muris* heart data, we identified six cell clusters (Fig. 4A) and compared them to biologically defined cell subpopulations such as cardiac muscle cells, endocardial cells, fibroblasts, and endothelial cells (Fig. 4B). We found that biologically defined cell subpopulations clustered together in the inferred transcription factor activity space, indicating that clustering the inferred transcription factor activity indeed reflects distinct biological functions of cell subpopulations. By identifying the most active transcription factors for each cluster, we further annotated the biological function of each cluster by the function of the top active transcription factors based on the importance scores. TAL1 was one of the most active transcription factors in clusters 3 and 5 (Fig. 4A; Supplemental Fig. S10A), whereas IRF8 was one of the most active transcription factors specific to cluster 6 (Fig. 4A; Supplemental Fig. S10B). These results were consistent with the biological cell labels (defined in the original data set), indicating that cells in cluster 3 were endothelial cells, cells in cluster 5 were endocardial cells, and cells in cluster 6 were leukocytes.

We performed the same analysis on the *Tabula Muris* brain data set. We identified eight clusters (Fig. 4C) and compared them to the biologically defined cell labels (Fig. 4D). The cells from the same biological cell type again clustered together in the inferred transcription factor activity analysis. NEUROD1 was among the most active transcription factors specific to cluster 2 (Fig. 4C; Supplemental Fig. S10C), whereas ASCL1 was one of the most active transcription factors specific to cluster 3 (Fig. 4C; Supplemental Fig. S10D). These results corresponded nicely to the biological cell labels, indicating that cells in cluster 2 were neurons and cells in cluster 3 were astrocytes (Fig. 4C,D). These findings indicate that BITFAM generates biologically significant profiles for individual cells and identifies cell clusters based on distinct inferred transcription factor activities.

To test whether the inferred transcription factor activities could be used for visualization and trajectory building of continuous cell populations, we analyzed the inferred transcription factor activities learned from the scRNA-seq analysis of hematopoietic differentiation. When visualizing the inferred transcription factor activities using UMAP, differentiation trajectories of common myeloid progenitors (CMPs) toward either megakaryocyte–erythroid progenitors (MEPs) or granulocyte-macrophage progenitors (GMPs) became apparent (Fig. 4E). We also applied the standard diffusion pseudo-time (DPT) approach (Haghverdi et al. 2015; Angerer et al. 2016) to the inferred transcription factor activities to generate a pseudo-time order and build a differentiation trajectory. When setting the common myeloid progenitors (CMPs) as start points, the BITFAM-DPT approach assigned the cells to two directions (Fig. 4F), underscoring the utility of BITFAM in building temporal trajectories.

### Comparing distinct transcription factor target genes as the input data for BITFAM

**Figure 5.**
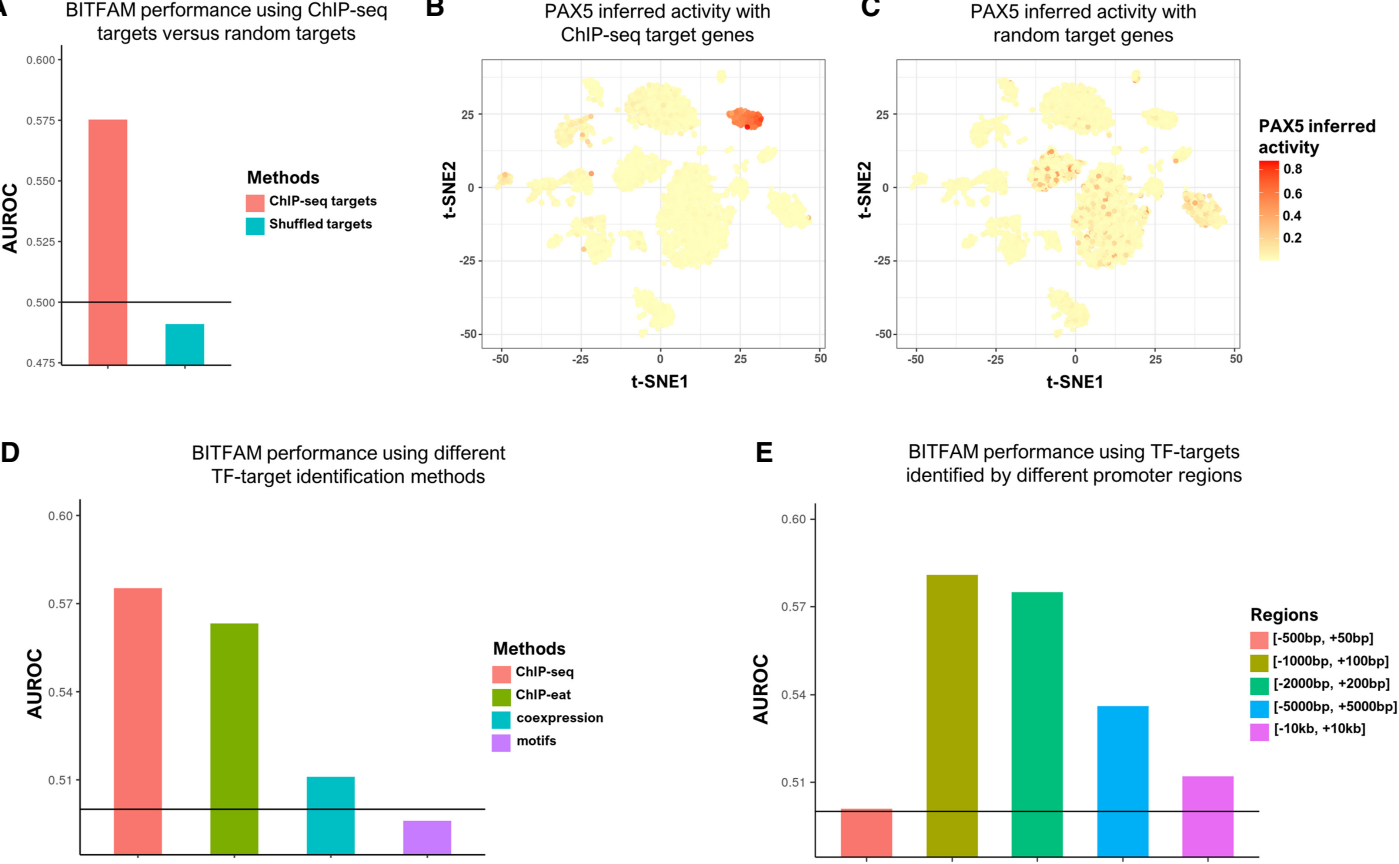
Performance of BITFAM when prior knowledge is varied. (*A*) The AUROC of transcription factor activities by shuffling ChIP-seq data. (*B*) The inferred activities of PAX5 in *Tabula Muris* lung data by ChIP-seq target genes. (*C*) The inferred activities of PAX5 in *Tabula Muris* lung data by shuffled target genes. (*D*) AUROC of transcription factor targets identification methods on CRISPRi data set. (*E*) AUROC of promoter region on CRISPRi data set.

To benchmark the performance of BITFAM using biological validation of a real-world scRNA-seq data, we used a scRNA-seq data set of CRISPRi studies in which 50 TFs were targeted for CRISPR-mediated deletion or knockdown (Genga et al. 2019). This data set is well-suited for assessing BITFAM performance because the depletion of TFs by CRISPR should result in lower TF activities. We applied BITFAM on the CRISPRi data set and used the AUROC based method to evaluate the performance (for details, see Methods). BITFAM infers transcription factor activities by incorporating prior knowledge of target genes that are predicted by ChIP-seq. To ascertain the importance of the ChIP-seq input data, we replaced ChIP-seq predicted target genes with randomly selected input genes and applied BITFAM to the CRISPRi and *Tabula Muris* lung data sets. We found that in the CRISPRi data, the AUROC for randomly selected transcription factor target genes drops down from 0.575 to 0.482 (Fig. 5A) and thus shows the necessity for selecting appropriate ChIP-seq-derived target genes. In the *Tabula Muris* lung data sets, the inferred activity of PAX5 in the original BITFAM model aligns nicely with the known B cell–specific function of PAX5; upon random shuffling of the target genes, the inferred activity of PAX5 is no longer specific to B cells, again highlighting the importance of the target genes (Fig. 5B,C).

We then examined the impact of how transcription factor target genes are predicted when using BITFAM. In addition to the ChIP-seq derived transcription factor target gene prior data, one could also use alternate approaches to predicting transcription factor target genes such as coexpression of TFs and genes, detecting transcription factor binding motifs in the promoter of genes, or combining ChIP-seq data with motif information such as ChIP-eat (Gheorghe et al. 2019) to generate TF-target gene sets. We benchmarked these methods of identifying transcription factor targets using the CRISPRi data set in the BITFAM framework and found that the default BITFAM ChIP-seq data prediction of target genes had the best performance (AUROC 0.575 for ChIP-seq target, AUROC 0.563 for ChIP-eat identified genes, AUROC 0.511 for coexpression genes, AUROC 0.496 for genes with binding motifs) (Fig. 5D). As distal regions identified by ChIP-seq can act as regulatory enhancers, we also evaluated the performance using distal ChIP-seq signals by extending the potential regulatory regions to the whole chromosome. The AUROC on CRISPRi data with this setting was 0.504 which was lower than the AUROC in the [−2000, +200] region (AUROC of 0.575) (Supplemental Fig. S11).

We then evaluated how the source of the ChIP-seq data could influence the results. BITFAM uses GTRD as the default database but there are also other ChIP-seq databases available such as ReMAP (Cheneby et al. 2020). Our evaluation showed that using a GTRD ChIP-seq-based approach performed better than when BITFAM was used with targets generated from the ReMAP database (Supplemental Fig. S12). We next asked the question whether the cell type from which the ChIP-seq data were generated was relevant for accurately inferring TF activities in various cell types. We therefore selectively removed ChIP-seq targets derived from certain cell types and assessed how the inferred TF activities were impacted. We specifically focused on selectively removing ChIP-seq data sets that may be of relevance to a given cell type (Supplemental Fig. S13). We found that for a transcription factor such as CEBPA, for which a large number of ChIP-seq data sets exist, BITFAM inference results of CEBPA activity match known biological functions of CEBPA in a hematopoietic development data set even when we used nonhematopoietic ChIP-seq data sets as prior knowledge (Supplemental Fig. S13A,B). However, when we removed non-B cell ChIP-seq data sets for the transcription factor PAX5, which is known to be highly active in B cells, we found that BITFAM was no longer able to accurately assign high PAX5 activity in B cells (Supplemental Fig. S13C,D). For the TF NEUROD1, there are currently 19 ChIP-seq data sets available, which have been derived from embryonic stem cells, pancreas beta cell, and the pituitary AtT-20 tumor cell line. None of these data sets include primary brain neurons; nevertheless, BITFAM inferred increased activity of NEUROD1 in brain neurons. However, when we removed the ChIP-seq data from the pituitary tumor line, which is arguably the closest to primary brain neurons, the BITFAM inference of increased NEUROD1 activity in selected populations was no longer associated with these cell types (Supplemental Fig. S13E,F). These data suggest that for certain TFs with increased activity in specific cell types, ChIP-seq data derived from those or related cell types may improve the inference of the TF activities.

When identifying the transcription factor targets from ChIP-seq data, the range of a gene promoter region could also conceivably influence the BITFAM output. The default approach for BITFAM is setting the promotor region as 2 kb upstream and 200 bp downstream from the transcription start site (TSS) of each gene. We then varied the promoter region length by comparing the BITFAM performance with the default setting to changing the regions as follows: (1) 500 bp upstream of TSS to 50 bp downstream from TSS [−500, +50], (2) 1000 bp upstream of TSS to 100 bp downstream from TSS [−1000, +100], (3) 5000 bp upstream of TSS to 5000 bp downstream from TSS [−5000, +5000], and (4) 10 kb upstream of TSS to 10 kb downstream from TSS [−10 kb, +10 kb]. We identified the ChIP-seq target genes on each of these different defined promoter regions and applied BITFAM using the CRISPRi data set. We found that setting the promoter region to [−1000, +100] and [−2000, +200] had the best performance (AUROC 0.581 for [−1000, +100], AUROC 0.575 for [−2000, +200], AUROC 0.536 for [−5000, +5000], AUROC 0.501 for [−500, +50], AUROC 0.512 for [−10 kb, +10 kb]) (Fig. 5E).

### Comparison of BITFAM with other methods to determine cell subpopulations and transcription factor activities

**Figure 6.**
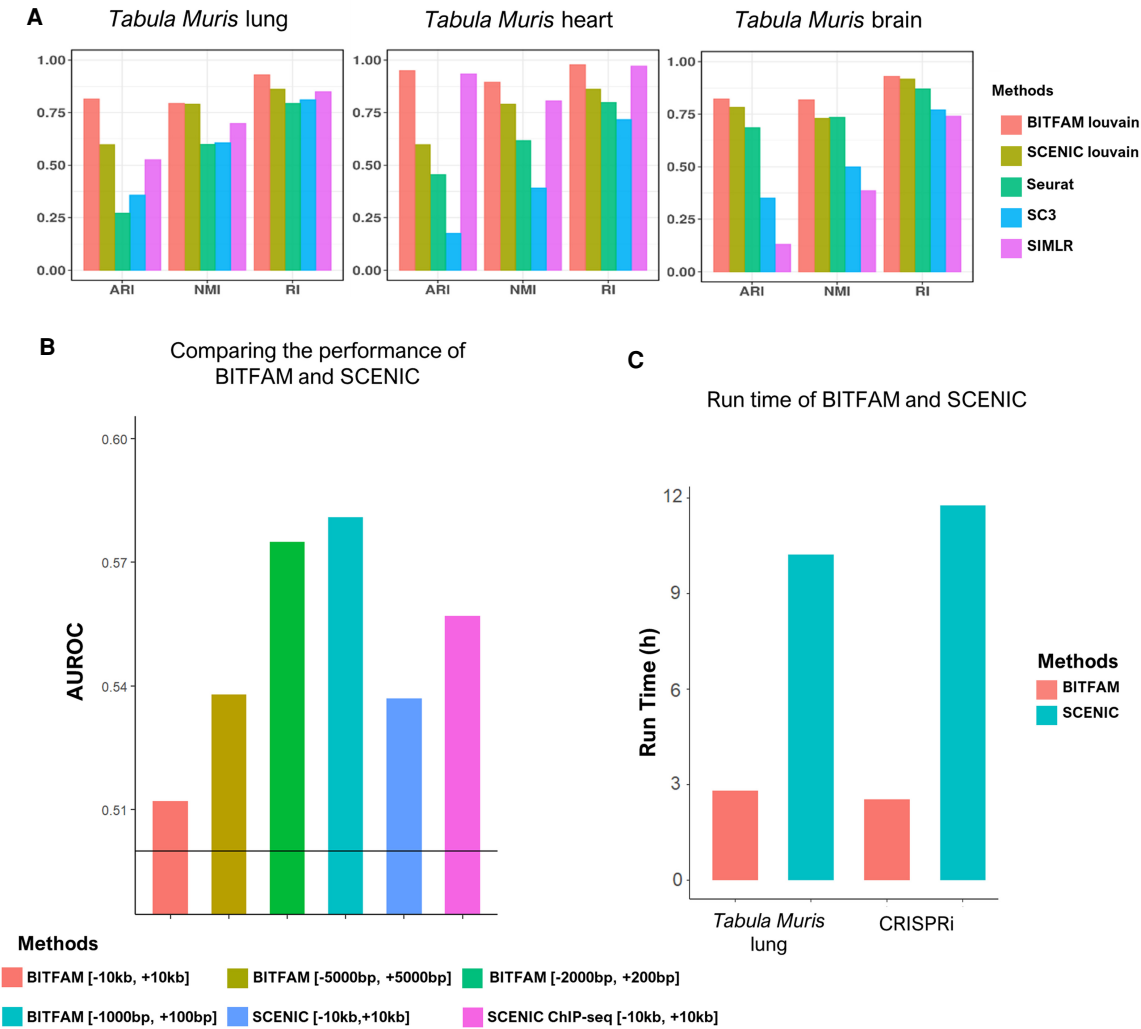
Comparison of BITFAM with other methods. (*A*) Clustering performance comparison between BITFAM + Louvain, SCENIC + Louvain, and three traditional clustering methods applied to the *Tabula Muris* lung, heart, and brain data sets. The clustering quality was evaluated based on three metrics: adjusted Rand index (ARI), Rand index (RI), and normalized mutual information (NMI). (*B*) Performance of BITFAM and SCENIC in the CRISPRi data. (*C*) The running time of BITFAM and SCENIC on the *Tabula Muris* lung data set and the CRISPRi data set.

We compared the clustering quality based on the inferred transcription factor activity profiles learned by BITFAM and SCENIC (Aibar et al. 2017) to the clustering results using other commonly used approaches such as Seurat (Butler et al. 2018), SIMLR (Wang et al. 2018), and SC3 (Kiselev et al. 2017). Cells were clustered using the inferred transcription factor activity profiles followed by applying Louvain's clustering algorithm (Blondel et al. 2008). The clustering quality was evaluated based on three metrics: adjusted Rand index (ARI), Rand index (RI), and normalized mutual information (NMI). In the *Tabula Muris* lung, heart, and brain data sets, the BITFAM-based clustering approach displayed improvements in terms of ARI, NMI, and RI when compared to other methods (Fig. 6A), thus highlighting the value of inferred transcription factor activity in the determination of cell subpopulations. The fact that clustering on inferred transcription factor activities segregates cells into previously established subpopulations with known distinct phenotypes and functions suggests that the BITFAM-inferred transcription factor activities have relevance for ascertaining cell function.

We next compared the overlap between transcription factor target genes identified by SCENIC and by BITFAM by focusing on the top-weighted genes using the same number of genes for a particular transcription factor. We found limited overlap between these two methods using the Jaccard index (Jaccard index > 0.1) (Supplemental Fig. S14; Supplemental Table S1), whereas SCENIC and BITFAM showed some overlap in the inferred transcription factor activities (Supplemental Fig. S15). We next also performed a GO enrichment analysis on the top 100 genes with the highest positive weights and compared this to the GO analysis on target genes identified from SCENIC. We found that BITFAM predicted target genes were more relevant to the known biological function of transcription factors. For example, RELB is an important transcription factor mediating inflammatory responses and cell survival (Baker et al. 2011). RELB target genes predicted by SCENIC were enriched for RNA or DNA metabolic processing (Supplemental Fig. S16A). On the other hand, the top-weighted target genes for RELB identified by BITFAM were enriched for processes such as immune cell activation, apoptosis, and proliferation (Supplemental Fig. S16B). There is a limited overlap of the transcription factor targets and pathways identified between the two methods, but the two methods also identify distinct target genes and pathways. The RELB ChIP-seq target genes were enriched for blood cell differentiation (Supplemental Fig. S17).

To directly compare the performance of BITFAM and SCENIC, we applied BITFAM and SCENIC on the CRISPRi data set. The AUROC of SCENIC in this data set using only motifs with 10 kb centered on the TSS ([−10 kb, +10 kb]) was 0.537. The AUROC of SCENIC using motifs and ChIP-seq data with 10 kb centered on TSS ([−10 kb, +10 kb]) was 0.557. These AUROCs were lower than the AUROCs of BITFAM using the optimal promoter regions we established for BITFAM (AUROC for BITFAM with region [−2000, +200] was 0.575; AUROC for BITFAM with region [−1000, +100] was 0.581). SCENIC-ChIP-seq performs better than BITFAM in the 5–10 kb regions (Fig. 6B; Supplemental Fig. S18). Even though the AUROC is widely used for the purpose of benchmarking, we also used a Precision-Recall AUC (PRAUC), which is more appropriate for unbalanced data sets such as the CRISPRi data in which only a small fraction of cells undergo TF deletion. Using the PRAUC, we again found that BITFAM performed slightly better than SCENIC when using the optimized regions for both methods (Supplemental Fig. S19). The run time of BITFAM on the *Tabula Muris* lung data set and CRISPRi data set was 2.81 h and 2.54 h, respectively, which was significantly lower than the corresponding run times of SCENIC using the same data sets (10.23 h and 11.78 h) and the same computer (AMD 3900XT 12-core CPU) (Fig. 6C).

We next compared the results of BITFAM to the CSHMM-TF model (Lin et al. 2020), which combines transcription factor activity inference with the generation of developmental trajectories based on a continuous state hidden Markov model. We applied the CSHMM-TF and BITFAM models in a lung development data set (Treutlein et al. 2014) and found that the inferred TFs activities from BITFAM overlapped with the results from the CSHMM-TF approach (Lin et al. 2020). For example, both BITFAM and CSHMM-TF inferred increased GATA6 activity in AT2 cells and increased SOX4/SOX5 activity in lung development (Supplemental Fig. S20), consistent with what is known about the mechanistic roles of these TFs in the respective cell types (Poncy et al. 2015; Flodby et al. 2017; Lin et al. 2020). However, the CSHMM-TF model also generates developmental trajectories, whereas BITFAM does not.

## Discussion

We have developed a model (BITFAM) for the inference of transcription factor activities in individual cells by analyzing scRNA-seq data and leveraging known transcription factor ChIP-seq data based on a Bayesian factor analysis. In contrast to many current methods developed for scRNA-seq data analysis that are primarily by the observed data itself, our approach of dimension reduction of individual cell gene expression profiles is guided by the integration of prior biological knowledge (ChIP-seq) with the observed data to infer transcription factor activities of the most variably expressed genes.

Our analysis showed that BITFAM can infer biologically meaningful transcription factor activities underlying distinct cell subpopulations. For example, TAL1 is an important factor for endothelial gene activation in vivo (Lazrak et al. 2004). It activates several endothelial-specific gene enhancers through essential E-box binding elements (De Val and Black 2009). BITFAM learned the high activity of TAL1 in endothelial cells, as shown in the analysis of the *Tabula Muris* lung data (Fig. 2). Similarly, our model inferred high PAX5 and EBF1 activity in B cells (Fig. 2; Supplemental Fig. S5) and high MAFB activity in macrophages (Fig. 2). In this context, it is important to note that *Pax*5 encodes the paired box 5 protein (human ortholog also known as BSAP), which is a specific regulator of the B lymphoid lineage (Nutt et al. 1999), whereas MAFB is an essential regulator of macrophages (Wu et al. 2016). In terms of the roles of inferred transcription factor activities in the setting of development and differentiation, GATA1 is a key factor in erythroid cell development (Takahashi et al. 1998), whereas CEBPA and STAT5A play important roles in granulocyte-macrophage progenitor development (Suh et al. 2006; Kimura et al. 2009). BITFAM inferred the activities of these transcription factors from the blood cell differentiation scRNA-seq data, which have been independently and experimentally proven to serve as key transcription factors of hematopoiesis. The regulatory roles of TFs in maintaining cell identity and cell function are highly complex, likely involving a network of TFs. We focused on selected TFs to illustrate that BITFAM can accurately infer the activities of selected well-established TFs in known biological functions of certain cell types. A more exciting application of BITFAM will be the future exploratory inference of transcription factor activities that have not yet been established in a given cell type, because it will generate novel testable hypotheses regarding putative regulatory roles of TFs in cell subpopulations. Such biological experiments in which individual TFs are deleted will also be necessary to test whether the negatively weighted genes identified by BITFAM indeed indicate putative repressive functions of the TFs.

It is important to point out that the mRNA expression levels of these transcription factors did not always match the known mechanistic roles of these factors in the cell lineage specification, and that BITFAM-inferred high activity of the selected transcription factors in cell subpopulations even when the measured mRNA expression levels of the transcription factors were minimal in the same cells (Fig. 2D,G; Supplemental Fig. S7). This could be because transcription factor activity is often regulated at the posttranslational level and does not require continuous de novo synthesis of mRNA (Zhao et al. 2018). These findings underscore the importance of integrating functional data into scRNA-seq data analysis to increase its functional and mechanistic significance.

Several methods have been developed to infer the transcription factor activities and build gene regulatory networks (GRN) from scRNA-seq data. The coexpression network is commonly used among these methods. The relationships of a transcription factor and its target genes are established by Pearson's correlation (Specht and Li 2017), mutual information (Chan et al. 2017), or machine learning models, such as random forest (Huynh-Thu et al. 2010; Aibar et al. 2017; Moerman et al. 2019) and deep neural network (Yuan and Bar-Joseph 2019) models. The transcription factor activities are then inferred from the coexpression network (Aibar et al. 2017). However, these models rely on the coexpression of the mRNA encoding for transcription factors, which can be a challenge because the activity of transcription factors is often regulated at a posttranslational level and key changes in activity may not be detected at the mRNA level. In addition, coexpression does not necessarily reflect a biologically meaningful relationship because it may be significantly influenced by noise in scRNA sequencing data (Freytag et al. 2015; Lähnemann et al. 2020).

We also compared BITFAM to SCENIC (Aibar et al. 2017), another recently developed transcription factor activity inference tool. SCENIC determines the target genes of a transcription factor based on gene coexpression coupled to a binding motif analysis, using a random forest model. SCENIC then combines feature importance with analysis of binding motifs, thus learning the inferred target genes of the analyzed transcription factors and corresponding inferred activity score for each transcription factor, in the data. Its key advantage is that it does not require prior ChIP-seq data and can thus be used for any transcription factor, especially those that have been recently identified and lack such prior knowledge. We found that SCENIC and BITFAM showed some overlap in the inferred transcription factor activities (Supplemental Fig. S15), but BITFAM outperforms SCENIC slightly in the CRISPRi validation data set when assessing transcription factors for which ChIP-seq data is available in the optimal searching regions (Fig. 5B; Supplemental Fig. S18). For our current performance comparisons, we used the AUROC approach that is commonly used in similar studies (Garcia-Alonso et al. 2019; Keenan et al. 2019; Nguyen et al. 2019; Holland et al. 2020). This method is based on the logFC of CRISPRi target TFs inferred activities between control cells and perturbed cells. It integrates all TFs together to generate one AUROC value that is reflective of overall performance.

One of the key challenges in the benchmarking of such TF inference algorithms is the limited availability of “ground truth” data sets. The CRISPRi data set was among the best we could find in terms of comprehensiveness, but one key limitation is that even if cells are transfected or transduced with guide RNAs to delete TFs, their deletion efficacy can vary and this can impact the validity of the comparison. For example, as shown in Supplemental Figure S18, when filtering for cells that expressed more than two copies of the guide RNA, we found an increase of the AUROC, thus suggesting that the overall low AUROC of both BITFAM and SCENIC may in part be related to that lack of definitive “ground truth data sets.” Our hope is that soon there will be more data sets available in which systematic deletions of multiple TFs are coupled to scRNA-seq, thus providing more opportunities for comparisons and development of algorithms with even greater accuracy of TF activity inference. That will also allow for more definitive conclusions regarding the comparative performance inference algorithms such as SCENIC and BITFAM, as well as newer algorithms developed in the future. One of the key advantages of BITFAM was its substantially lower run time (∼60%–70% lower run time than SCENIC) (Fig. 6C).

One possible application of BITFAM would be the discovery of novel heterogeneous subpopulations that cannot be identified using standard clustering approaches because inferred transcription factor activities may identify more subtle phenotype differences owing to the regulatory function of transcription factors than only clustering on global gene expression. Our goal in the current study was to establish the value of combining ChIP-seq data and Bayesian analysis for inferring transcription factor activities using known transcription factors in known cell types. We used clustering and comparisons to the identity of biologically established cell subpopulations (which are likely distinct in their transcription factor activity profiles) merely as an approach to validate BITFAM. Establishing additional novel downstream approaches for transcription factor–based trajectory building or identifying transcription factor activity–based subclusters would be an exciting future application of BITFAM. BITFAM can be readily combined with multiple clustering or trajectory building approaches. The CSHMM-TF (Lin et al. 2020) combines transcription factor activity inference with the generation of developmental trajectories based on a continuous state hidden Markov model. Although the CSHMM-TF approach is ideally suited for temporal or developmental trajectories involving state transitions, BITFAM can infer transcription factor activities for data sets that do not contain temporal trajectories and state transitions, thus complementing CSHMM-TF.

Another important consideration for using BITFAM is the choice of the input ChIP-seq data. We found that casting a wider net of potential regulatory ChIP-seq targets by choosing a larger promoter region did not improve the performance of BITFAM (Fig. 5E; Supplemental Fig. S12). We acknowledge that limiting the promoter region to [−2000, +200] bp around the TSS may exclude important distal enhancer and regulatory regions. A recent study with scATAC-seq data has shown that a large proportion of accessible chromatin regions can be more than 10 kb distant from the nearest TSS (Domcke et al. 2020), thus underscoring the potential role of distant regulatory regions. Matching distant regulatory regions to the putative target genes remains an active area of research. One possible approach could be the future integration of bulk and single-cell Hi-C data that could provide additional information on the most relevant distant regulatory regions. Growing consensus on identifying putative distal regulatory regions could allow for this information to be incorporated into BITFAM or analogous Bayesian models in the future.

In regard to the quality and extent of the ChIP-seq data that is required to establish the prior knowledge in a Bayesian inference model, one finds a wide variety in the number of ChIP-seq data sets for any individual transcription factor (usually a function of how many groups are studying the transcription factor). Furthermore, existing ChIP-seq data are generated from bulk cells or tissues, and it is unclear whether the ChIP-seq data derived from one cell type is as relevant for a scRNA-seq experiment as one that is derived from another cell or tissue type. We found that for certain transcription factors such as NEUROD1 and PAX5 for which BITFAM inferred increased activities in neurons (NEUROD1) and B Cells (PAX5), the presence of ChIP-seq data sets derived from related cell types was required to allow for these specific inferences in the analyzed scRNA-seq data. However, for transcription factors such as CEBPA, removal of selected ChIP-seq data sets did not significantly impact the inferred activities. This could indicate the importance of having a larger number of input data sets (such as the case for CEBPA) to create a robust prior knowledge required for a successful Bayesian prediction model or it could also reflect that some transcription factors such as CEBPA may have similar ChIP-seq targets across tissues and cells, whereas other transcription factors such as NEUROD1 and PAX5 may be associated with cell type–specific targets. These limitations of applying bulk ChIP-seq data derived from several cell types to a given scRNA-seq data set derived from a different group of cell types may in part be overcome in the future with the emerging availability of context-specific single-cell chromatin accessibility data as seen in studies of the transcription factor targets and regulatory mechanism by scATAC-seq (Buenrostro et al. 2015; Cusanovich et al. 2018; Duren et al. 2018; Jia et al. 2018). In the future as more and more scATAC-seq analyses are performed concomitantly with scRNA-seq analyses, we may improve BITFAM by incorporating the scATAC-seq data into the Bayesian hierarchical model to have more accurate and specific results. BITFAM allows users to filter TF ChIP-seq target genes based on the accessibility data provided by ATAC-seq data, but there are also opportunities for future iterations of BITFAM or related Bayesian approaches to incorporate scATAC-seq data into the Bayesian hierarchical model itself, thus narrowing down the potential ChIP-seq targets by intersecting them with the open chromatin regions in a cell type–specific manner and thus improving the quality of the inference.

The idea of integrating prior biological knowledge with RNA-seq data to infer gene regulatory network has also been explored in several previous approaches (James et al. 2010; Arrieta-Ortiz et al. 2015; Ji et al. 2019; Miraldi et al. 2019; Jackson et al. 2020). These methods were developed for specific cell types and treated the prior knowledge as a fixed network, thus only learning the interactions within this network. Such approaches could be very helpful when there are established specific TF–gene relationships in a given cell type. In contrast, BITFAM assigns a probability to the TF–gene interaction without requiring cell-specific prior knowledge and provides a generalized approach to identify novel target genes for transcription factors in the specific data set.

TF activities inferred by BITFAM can be used to identify putative cell type–specific TFs. We chose a random forest model to generate such cell type–specific TF profiles but other methods can also be applied for such an analysis. For example, a linear model could be used to identify differentally activated transcription factors across cell types. We posited that the ranked feature list in the random forest model allows for the evaluation of the activity of each TF in the presence of other TFs and their potential nonlinear relationships, whereas ranking based on the scale of mean *Z*-values, which would only provide a univariate analysis. However, users retain the flexibility to choose the downstream analysis method that may be best suited for establishing cell type–specific TFs based on BITFAM-inferred activities.

In summary, we have developed a Bayesian factor analysis model to infer transcription factor activity in individual cells, and we use this approach to develop key biological hypotheses regarding the regulatory transcription factors in each cell as well as derive insights into the biological functions of cell subpopulations.

## Methods

### The Bayesian inference transcription factor activity model (BITFAM)

BITFAM is a model of Bayesian factor analysis (Lopes and West 2004) that aims at the decomposition of scRNA-seq profiles *Y*: *Y* = *WZ* + Φ. BITFAM assumes a linear relationship between the scRNA-seq data and the inferred transcription factor activities specified by *Z* and the transcription factor–specific gene regulation patterns specified by *W*. Here, we describe the structure and relation of *Y*, *W*, and *Z*, and we define the probability distributions for the matrix elements.

Matrix *W* is the weight matrix that represents the evidence of transcription factors and their target genes obtained from the transcription factor ChIP-seq data. We assign normal prior distributions on elements of matrix *W* with two different variances as follows.

We assume that the weight of a transcription factor *k* on gene *n* follows normal distribution that is modeled with the prior distribution for *W*_*nk*_:

$$W_{nk}∼\{\begin{array}{l} N\left(W_{nk}|0,1/\delta_{k}\right)\;\;\;\mathrm{if}\;\mathrm{gene}\;n\;\mathrm{is}\;\mathrm{targeted}\;\mathrm{by}\;\mathrm{transcription}\;\mathrm{factor}\;k\;\\ N(W_{nk}|0,\;0.001)\;\;\;\mathrm{otherwise}\; \end{array}$$

for gene *n* = 1, …, *N* and transcription factor *k* = 1, …, *K*. We use automatic relevance determination (ARD) to model the parameter *δ*_*k*_, i.e.,

$$\delta_{k}∼\mathrm{Gamma}(1\times 10^{-3},\;1\times 10^{-3}).$$

In the target gene list determined by the ChIP-seq data, some of the genes may not be the targets of the transcription factors in a given scRNA-seq data set because the same transcription factor may have different targets in distinct cell subtypes. It is therefore necessary to develop a method to automatically infer which target genes are relevant and switch the other genes off. The automatic relevance determination (ARD) (MacKay 1996) is used for this purpose.

Matrix *Z* is facilitated as the transcription factor activities for each cell in our model. We assign a Beta prior to the activity of transcription factor *k* in *m*th cell (*m* = 1, …, *M*), that is,

$$Z_{km}∼Beta(0.5,\;0.5).$$

The residual noise *ϕ*_*nm*_ is modeled by a normal distribution with variance *ε*. $\phi_{nm}∼N(0,\;\varepsilon );$ ε∼Gamma(1,1)forgenen.

The likelihood of our BITFAM is

Y|(W,Z,Φ)∼Normal(WZ, Φ).

The examples of posterior distribution of 1/*σ*_*k*_ and weights can be found in Supplemental Figure S21.

### Parameter inference

To achieve scalability to large numbers of cells and genes, we use approximate Bayesian inference based on variational methods (Ghahramani and Matthew 2000; Wainwright and Jordan 2008). The variational method is to infer the posterior distributions over all unobserved variables using a factorized form. The final weight matrix *W* and the inferred transcription factor activities matrix *Z* are constructed by taking the means of the samples from the posterior distributions. The sample size is 300.

We implemented the inference of BITFAM with R (R Core Team 2020) package Rstan (Version 2.18.2). Rstan implements an automatic variational inference algorithm, called Automatic Differentiation Variational Inference (ADVI) (Kucukelbir et al. 2017). ADVI uses Monte Carlo integration to approximate the variational objective function, the ELBO (evidence lower bound). Stochastic gradient ascent is used to optimize the ELBO in the real-coordinate space. The algorithm stops when the mean change of ELBO is below 0.01.

### Processing and analysis of the scRNA-seq data sets

The *Tabula Muris* lung, heart, and brain data were generated from Smart-seq2 (5447, 4321, and 6315 cells in each organ) and processed. The Seurat R objects with raw counts are downloaded from figshare (https://figshare.com/projects/Tabula_Muris_Transcriptomic_characterization_of_20_organs_and_tissues_from_Mus_musculus_at_single_cell_resolution/27733). The cell types were labeled in each organ by the expression of well-known marker genes (*Tabula Muris* et al. 2018). The blood cell development expression data generated on the MARS-seq platform (10,368 cells in total) were obtained from NCBI Gene Expression Omnibus (GEO; https://www.ncbi.nlm.nih.gov/geo/) (accession GSE72857). Using FACS, 2729 myeloid progenitor cells (CMP, GMP, and MEP) were identified.

We used the NormalizeData function from R package Seurat (Butler et al. 2018) to normalize the gene expression in each cell. We chose the normalization method “LogNormalize,” which normalizes the feature expression measurements for each cell by the total expression, multiplied by a scale factor (10,000 by default). The normalized data were log-transformed.

We identified the most variably expressed genes using the function FindVariableGenes in Seurat. It calculates the average expression and dispersion for each gene, places these genes into bins, and then calculates a *Z*-score for dispersion within each bin (Butler et al. 2018). We defined the most variable expressed genes with the following cutoff: the mean log-transformed expression of genes across all cells should be higher than 0.1, and the variance to mean ratio should be larger than 1. The summary of model input of each data set is provided in the Supplemental Files (Supplemental Fig. S1).

### The identification of transcription factor target genes

The ChIP-seq data were obtained from the Gene Transcription Regulation Database (GTRD v19.04) (Yevshin et al. 2019), which is a database of transcription factor binding sites for human and mouse obtained from the uniformly processed ChIP-seq data. We downloaded the meta clusters intervals that have been integrated from the ChIP-seq data peak intervals from different projects and peak calling tools. For each transcription factor, we defined its target genes by overlapping the ChIP-seq peak intervals in the promotor region of the genes. We set the promotor region as 2 kb upstream and 200 bp downstream from the transcription starting site (TSS) of each gene. The information of the TSS was downloaded from the UCSC Table Browser (Karolchik et al. 2004).

For each data set, we selected the expressed transcription factors as input to our model. The transcription factor prior target genes are the intersection between ChIP-seq-based regulatory target genes and the most variably expressed genes in each scRNA-seq data set. We excluded transcription factors with less than 10 (prior) target genes.

### Random forest model to identify the marker transcription factors for specific cell types

For each cell type in a data set, we label the cells for a given cell type as 1 and the other cells as 0. The inferred transcription factors activities (*Z* matrix) are the features as input. We train the random forest model with the R package randomForest (version 4.6). Then for each type, we identify the most significant transcription factors by the feature importance score.

### Louvain's algorithm

Louvain's algorithm detects communities in a graph by maximizing a modularity score for each community, where the modularity is the density of nodes within that community (Blondel et al. 2008). Louvain's algorithm can also be used for clustering. To construct a graph suitable for Louvain's algorithm, we built a fully connected graph of all cells based on the inferred transcription factor activities in cells. The edge weights between nodes (cells) are diffusion distances and computed using the R package destiny (Angerer et al. 2016). The top 20% smallest weight edges, that is, the 20% closest distances between cells were used to form a new graph and we applied Louvain's algorithm. We used the R package igraph (Version 1.2.4.1) to build the graph and implemented Louvain's algorithm.

### Clustering quality metrics

#### Rand index (RI) and adjusted Rand index (ARI)

Given a set of elements and two classifications of these elements, the RI is defined as

$$RI=\frac{a+b}{\left(\begin{array}{c} n\\ 2 \end{array}\right)}$$

where *a* refers to the number of times a pair of elements belongs to the same cluster across the two classifications, and *b* refers to the number of times a pair of elements are in different clusters across the two classifications (Hubert and Arabie 1985). The RI represents the frequency of occurrence of agreements over the total pairs, and ranges between 0 and 1. When the two classifications are agreed perfectly, the Rand index is 1.

The overlap between the two classifications can be presented by a contingency table, in which each entry denotes the number of objects shared between the two classifications. The ARI is defined as

$$\mathrm{ARI}=\frac{\sum_{ij}\left(\begin{array}{c} n_{ij}\\ 2 \end{array}\right)-\left[\sum_{i}\left(\begin{array}{c} a_{i}\\ 2 \end{array}\right)\sum_{j}\left(\begin{array}{c} b_{j}\\ 2 \end{array}\right)\right]/\left(\begin{array}{c} n\\ 2 \end{array}\right)}{\frac{1}{2}\left[\sum_{i}\left(\begin{array}{c} a_{i}\\ 2 \end{array}\right)+\sum_{j}\left(\begin{array}{c} b_{j}\\ 2 \end{array}\right)\right]-\left[\sum_{i}\left(\begin{array}{c} a_{i}\\ 2 \end{array}\right)\sum_{j}\left(\begin{array}{c} b_{j}\\ 2 \end{array}\right)\right]/\left(\begin{array}{c} n\\ 2 \end{array}\right)}$$

where *n*_*ij*_ is the value in the contingency table; *a*_*i*_ is the sum of the *i*th row of the contingency table; and *b*_*j*_ is the sum of the *j*th column of the contingency table. The ARI corrects the RI for chance (Hubert and Arabie 1985).

#### Normalized mutual information (NMI)

Given a set of *n* elements and a classification *X* of these elements, and a clustering result *Y*, the Normalized Mutual Information (NMI) is defined as

$$\mathrm{NMI}=\frac{2\times I(X;Y)}{\left[H(X)+H(Y)\right]}$$

where *H*(.) is the entropy of class labels; $H(.)=\;\sum_{i}(-p_{i})\log(\;p_{i})$; *p*_*i*_ is the probability of an element belonging to cluster *i*; and *I*(*X*;*Y*) is the mutual information defined as *I*(*X*;*Y*) = *H*(*X*) − *H*(*X*|*Y*).

### Jaccard index

Jaccard index is a statistic used for quantifying the similarity between two sets. It is defined as $J(A,B)=\frac{\left|A\cap B\right|}{\left|A\cup B\right|}$, where *A* and *B* are sets.

### Diffusion pseudo time (DPT)

Diffusion pseudo time (DPT) was calculated using diffusion distances between each cell based on the profiles of reduced dimensions (PCA or BITFAM). One CMP cell served as the starting point, whereas GMP and MEP cells were the end points of each branch. Pseudo time toward MEP and GMP branches was scaled between [0, 1] and [0, −1] to show the branching more distinctly. The R package destiny (Version 2.14.0) was used to compute the Diffusion pseudo time (DPT) (Angerer et al. 2016).

### Other clustering approaches

Seurat (Version 3.0), SIMLR (Version 1.10.0), and SC3 (Version 1.12.0) were downloaded from Bioconductor (Version 3.9). Seurat was run with default parameters and set 20 as the number of principal components used for clustering. SIMLR was run with default parameters and the number of clusters was detected by the function SMLR::SIMLR_Estimate_Number_of_Clusters. SC3 was run with default parameters and the number of clusters was detected by the function SC3::sc3_estimate_k.

### Gene Ontology enrichment analysis

The Gene Ontology enrichment analysis of the top inferred weighted genes for transcription factors was conducted using The Database for Annotation, Visualization and Integrated Discovery (DAVID) v6.8 (Huang et al. 2009) (adjusted *P*-value < 0.005). We used the level 5 Biological Process (BP) as the annotation of sets to annotate the function of the selected genes.

### Benchmarking with AUROC on CRISPRi data

The CRISPRi data include 141 perturbation experiments with CRISPR-targeting of 50 transcription factors. It is available on NCBI GEO (accession GSE127202). For each CRISPRi perturbation experiment, we calculated the logFC of all transcription factor inferred activities comparing perturbed cells and control cells. For example, for CRISPR-targeting of TF *X*, we calculated the logFC of the inferred activities of all TFs in cells in which *X* was perturbed versus the inferred activities of the TFs in cells in which *X* was not perturbed. The positive class (perturbed cells) and negative class (nonperturbed cells) represent the biological ground truth that is determined by the CRISPRi experiment. So, a thresholding of logFC of inferred *X* activity from BITFAM in all cells will determine whether a cell will be inferred as *X* deletion (lower logFC). When this threshold varies, then an AUROC can be computed. For every TF, we used the same approach, thus allowing us to calculate an overall AUROC for all TFs. This AUROC indicates how accurately inferred TF activities were associated with the positive and negative classes.

We performed the AUROC analysis with the R package yardstick (version 0.0.3).

### Other transcription factor activity inference approaches using CRISPRi data

The genes with the top 500 highest (absolute value) correlations (*P*-value < 0.05) were defined as coexpressed genes of the transcription factor. For transcription factor binding motifs on promoters, we chose the region from 2000 bp upstream of the transcription starting site (TSS) to 200 bp downstream from TSS as the promoter. Then we used a motif scanning tool, FIMO (Grant et al. 2011), to search the transcription factor motifs from HOCOMOCO (Kulakovskiy et al. 2018) and JASPAR (Fornes et al. 2020) on the gene promoters. The genes with binding motifs (*P*-value < 0.0001) were identified as the targets of TFs. For the ChIP-eat method, it combines computational transcription factor binding models and ChIP-seq peaks to automatically predict direct TF–DNA interactions. We downloaded the Individual BED files for specific TFs on the UniBind website (https://unibind.uio.no).

### Software availability

The BITFAM (version 1.2.0) is implemented in R (R Core Team 2020), and the source code is available at Supplemental Code. BITFAM (version 1.2.0) can be freely downloaded from GitHub (https://github.com/jaleesr/BITFAM).

## Supplementary Material

Supplemental Material

## References

[GR265595GAOC1] Aibar S, González-Blas CB, Moerman T, Huynh-Thu VA, Imrichova H, Hulselmans G, Rambow F, Marine JC, Geurts P, Aerts J, 2017. SCENIC: single-cell regulatory network inference and clustering. Nat Methods 14: 1083–1086. 10.1038/nmeth.4463 28991892 PMC5937676

[GR265595GAOC2] Amir ED, Davis KL, Tadmor MD, Simonds EF, Levine JH, Bendall SC, Shenfeld DK, Krishnaswamy S, Nolan GP, Pe'er D. 2013. viSNE enables visualization of high dimensional single-cell data and reveals phenotypic heterogeneity of leukemia. Nat Biotechnol 31: 545–552. 10.1038/nbt.2594 23685480 PMC4076922

[GR265595GAOC3] Angerer P, Haghverdi L, Büttner M, Theis FJ, Marr C, Buettner F. 2016. *Destiny*: diffusion maps for large-scale single-cell data in R. Bioinformatics 32: 1241–1243. 10.1093/bioinformatics/btv715 26668002

[GR265595GAOC4] Arrieta-Ortiz ML, Hafemeister C, Bate AR, Chu T, Greenfield A, Shuster B, Barry SN, Gallitto M, Liu B, Kacmarczyk T, 2015. An experimentally supported model of the *Bacillus subtilis* global transcriptional regulatory network. Mol Syst Biol 11: 839. 10.15252/msb.20156236 26577401 PMC4670728

[GR265595GAOC5] Bai J, Li K. 2012. Statistical analysis of factor models of high dimension. Ann Stat 40: 436–465.

[GR265595GAOC6] Baker RG, Hayden MS, Ghosh S. 2011. NF-κB, inflammation, and metabolic disease. Cell Metab 13: 11–22. 10.1016/j.cmet.2010.12.008 21195345 PMC3040418

[GR265595GAOC7] Blondel VD, Guillaume JL, Lambiotte R, Lefebvre E. 2008. Fast unfolding of communities in large networks. J Stat Mech: Theory Exp 2008: P10008. 10.1088/1742-5468/2008/10/P10008

[GR265595GAOC8] Buenrostro JD, Wu B, Litzenburger UM, Ruff D, Gonzales ML, Snyder MP, Chang HY, Greenleaf WJ. 2015. Single-cell chromatin accessibility reveals principles of regulatory variation. Nature 523: 486–490. 10.1038/nature14590 26083756 PMC4685948

[GR265595GAOC9] Buettner F, Pratanwanich N, McCarthy DJ, Marioni JC, Stegle O. 2017. f-scLVM: scalable and versatile factor analysis for single-cell RNA-seq. Genome Biol 18: 212. 10.1186/s13059-017-1334-8 29115968 PMC5674756

[GR265595GAOC10] Butler A, Hoffman P, Smibert P, Papalexi E, Satija R. 2018. Integrating single-cell transcriptomic data across different conditions, technologies, and species. Nat Biotechnol 36: 411–420. 10.1038/nbt.4096 29608179 PMC6700744

[GR265595GAOC11] Chan TE, Stumpf MPH, Babtie AC. 2017. Gene regulatory network inference from single-cell data using multivariate information measures. Cell Syst 5: 251–267.e3. 10.1016/j.cels.2017.08.014 28957658 PMC5624513

[GR265595GAOC12] Cheneby J, Menetrier Z, Mestdagh M, Rosnet T, Douida A, Rhalloussi W, Bergon A, Lopez F, Ballester B. 2020. ReMap 2020: a database of regulatory regions from an integrative analysis of Human and Arabidopsis DNA-binding sequencing experiments. Nucleic Acids Res 48: D180–D188. 10.1093/nar/gkz945 31665499 PMC7145625

[GR265595GAOC13] Cusanovich DA, Reddington JP, Garfield DA, Daza RM, Aghamirzaie D, Marco-Ferreres R, Pliner HA, Christiansen L, Qiu X, Steemers FJ, 2018. The *cis*-regulatory dynamics of embryonic development at single-cell resolution. Nature 555: 538–542. 10.1038/nature25981 29539636 PMC5866720

[GR265595GAOC14] De Val S, Black BL. 2009. Transcriptional control of endothelial cell development. Dev Cell 16: 180–195. 10.1016/j.devcel.2009.01.014 19217421 PMC2728550

[GR265595GAOC15] Domcke S, Hill AJ, Daza RM, Cao J, O'Day DR, Pliner HA, Aldinger KA, Pokholok D, Zhang F, Milbank JH, 2020. A human cell atlas of fetal chromatin accessibility. Science 370: eaba7612. 10.1126/science.aba7612 33184180 PMC7785298

[GR265595GAOC16] Duren Z, Chen X, Zamanighomi M, Zeng W, Satpathy AT, Chang HY, Wang Y, Wong WH. 2018. Integrative analysis of single-cell genomics data by coupled nonnegative matrix factorizations. Proc Natl Acad Sci 115: 7723–7728. 10.1073/pnas.1805681115 29987051 PMC6065048

[GR265595GAOC17] Flodby P, Li C, Liu Y, Wang H, Rieger ME, Minoo P, Crandall ED, Ann DK, Borok Z, Zhou B. 2017. Cell-specific expression of aquaporin-5 (*Aqp5*) in alveolar epithelium is directed by GATA6/Sp1 via histone acetylation. Sci Rep 7: 3473. 10.1038/s41598-017-03152-7 28615712 PMC5471216

[GR265595GAOC18] Fornes O, Castro-Mondragon JA, Khan A, van der Lee R, Zhang X, Richmond PA, Modi BP, Correard S, Gheorghe M, Baranasic D, 2020. JASPAR 2020: update of the open-access database of transcription factor binding profiles. Nucleic Acids Res 48: D87–D92. 10.1093/nar/gkz1001 31701148 PMC7145627

[GR265595GAOC19] Freytag S, Gagnon-Bartsch J, Speed TP, Bahlo M. 2015. Systematic noise degrades gene coexpression signals but can be corrected. BMC Bioinformatics 16: 309. 10.1186/s12859-015-0745-3 26403471 PMC4583191

[GR265595GAOC20] Garcia-Alonso L, Holland CH, Ibrahim MM, Turei D, Saez-Rodriguez J. 2019. Benchmark and integration of resources for the estimation of human transcription factor activities. Genome Res 29: 1363–1375. 10.1101/gr.240663.118 31340985 PMC6673718

[GR265595GAOC21] Genga RMJ, Kernfeld EM, Parsi KM, Parsons TJ, Ziller MJ, Maehr R. 2019. Single-cell RNA-sequencing-based CRISPRi screening resolves molecular drivers of early human endoderm development. Cell Rep 27: 708–718.e10. 10.1016/j.celrep.2019.03.076 30995470 PMC6525305

[GR265595GAOC22] Ghahramani Z, Matthew JB. 2000. Variational inference for Bayesian mixtures of factor analysers. Adv Neural Inf Process Syst 12: 449–455.

[GR265595GAOC23] Gheorghe M, Sandve GK, Khan A, Chèneby J, Ballester B, Mathelier A. 2019. A map of direct TF–DNA interactions in the human genome. Nucleic Acids Res 47: 7715. 10.1093/nar/gkz582 31251803 PMC6698730

[GR265595GAOC24] Grant CE, Bailey TL, Noble WS. 2011. FIMO: scanning for occurrences of a given motif. Bioinformatics 27: 1017–1018. 10.1093/bioinformatics/btr064 21330290 PMC3065696

[GR265595GAOC25] Haghverdi L, Buettner F, Theis FJ. 2015. Diffusion maps for high-dimensional single-cell analysis of differentiation data. Bioinformatics 31: 2989–2998. 10.1093/bioinformatics/btv325 26002886

[GR265595GAOC26] Hand D. 2013. Latent variable models and factor analysis: a unified approach, 3rd ed. (ed. Bartholomew DJ, ). Wiley, Chichester, UK.

[GR265595GAOC27] Holland CH, Tanevski J, Perales-Patón J, Gleixner J, Kumar MP, Mereu E, Joughin BA, Stegle O, Lauffenburger DA, Heyn H, 2020. Robustness and applicability of transcription factor and pathway analysis tools on single-cell RNA-seq data. Genome Biol 21: 36. 10.1186/s13059-020-1949-z 32051003 PMC7017576

[GR265595GAOC28] Huang DW, Sherman BT, Lempicki RA. 2009. Bioinformatics enrichment tools: paths toward the comprehensive functional analysis of large gene lists. Nucleic Acids Res 37: 1–13. 10.1093/nar/gkn923 19033363 PMC2615629

[GR265595GAOC29] Hubert L, Arabie P. 1985. Comparing partitions. J Classif 2: 193–218. 10.1007/BF01908075

[GR265595GAOC30] Huynh-Thu VA, Irrthum A, Wehenkel L, Geurts P. 2010. Inferring regulatory networks from expression data using tree-based methods. PLoS One 5: e12776. 10.1371/journal.pone.0012776 20927193 PMC2946910

[GR265595GAOC31] Jackson CA, Castro DM, Saldi GA, Bonneau R, Gresham D. 2020. Gene regulatory network reconstruction using single-cell RNA sequencing of barcoded genotypes in diverse environments. eLife 9: e51254. 10.7554/eLife.51254 31985403 PMC7004572

[GR265595GAOC32] James GM, Sabatti C, Zhou N, Zhu J. 2010. Sparse regulatory networks. Ann Appl Stat 4: 663–686. 10.1214/10-AOAS350 21625366 PMC3102251

[GR265595GAOC33] Ji Z, He L, Regev A, Struhl K. 2019. Inflammatory regulatory network mediated by the joint action of NF-kB, STAT3, and AP-1 factors is involved in many human cancers. Proc Natl Acad Sci 116: 9453–9462. 10.1073/pnas.1821068116 30910960 PMC6511065

[GR265595GAOC34] Jia G, Preussner J, Chen X, Guenther S, Yuan X, Yekelchyk M, Kuenne C, Looso M, Zhou Y, Teichmann S, 2018. Single cell RNA-seq and ATAC-seq analysis of cardiac progenitor cell transition states and lineage settlement. Nat Commun 9: 4877. 10.1038/s41467-018-07307-6 30451828 PMC6242939

[GR265595GAOC35] Jung M, Wells D, Rusch J, Ahmad S, Marchini J, Myers SR, Conrad DF. 2019. Unified single-cell analysis of testis gene regulation and pathology in five mouse strains. eLife 8: e43966. 10.7554/eLife.43966 31237565 PMC6615865

[GR265595GAOC36] Karolchik D, Hinrichs AS, Furey TS, Roskin KM, Sugnet CW, Haussler D, Kent WJ. 2004. The UCSC Table Browser data retrieval tool. Nucleic Acids Res 32: D493–D496. 10.1093/nar/gkh103 14681465 PMC308837

[GR265595GAOC37] Keenan AB, Torre D, Lachmann A, Leong AK, Wojciechowicz ML, Utti V, Jagodnik KM, Kropiwnicki E, Wang Z, Ma'ayan A. 2019. ChEA3: transcription factor enrichment analysis by orthogonal omics integration. Nucleic Acids Res 47: W212–W224. 10.1093/nar/gkz446 31114921 PMC6602523

[GR265595GAOC38] Kimura A, Rieger MA, Simone JM, Chen W, Wickre MC, Zhu BM, Hoppe PS, O'Shea JJ, Schroeder T, Hennighausen L. 2009. The transcription factors STAT5A/B regulate GM-CSF-mediated granulopoiesis. Blood 114: 4721–4728. 10.1182/blood-2009-04-216390 19779039 PMC2780307

[GR265595GAOC39] Kiselev VY, Kirschner K, Schaub MT, Andrews T, Yiu A, Chandra T, Natarajan KN, Reik W, Barahona M, Green AR, 2017. SC3: consensus clustering of single-cell RNA-seq data. Nat Methods 14: 483–486. 10.1038/nmeth.4236 28346451 PMC5410170

[GR265595GAOC40] Kiselev VY, Andrews TS, Hemberg M. 2019. Challenges in unsupervised clustering of single-cell RNA-seq data. Nat Rev Genet 20: 273–282. 10.1038/s41576-018-0088-9 30617341

[GR265595GAOC41] Kucukelbir A, Tran D, Ranganath R, Gelman A, Blei DM. 2017. Automatic differentiation variational inference. J Mach Learn Res 18: 430–474.

[GR265595GAOC42] Kulakovskiy IV, Vorontsov IE, Yevshin IS, Sharipov RN, Fedorova AD, Rumynskiy EI, Medvedeva YA, Magana-Mora A, Bajic VB, Papatsenko DA, 2018. HOCOMOCO: towards a complete collection of transcription factor binding models for human and mouse via large-scale ChIP-Seq analysis. Nucleic Acids Res 46: D252–D259. 10.1093/nar/gkx1106 29140464 PMC5753240

[GR265595GAOC43] Lähnemann D, Köster J, Szczurek E, McCarthy DJ, Hicks SC, Robinson MD, Vallejos CA, Campbell KR, Beerenwinkel N, Mahfouz A, 2020. Eleven grand challenges in single-cell data science. Genome Biol 21: 31. 10.1186/s13059-020-1926-6 32033589 PMC7007675

[GR265595GAOC44] Lazrak M, Deleuze V, Noel D, Haouzi D, Chalhoub E, Dohet C, Robbins I, Mathieu D. 2004. The bHLH TAL-1/SCL regulates endothelial cell migration and morphogenesis. J Cell Sci 117: 1161–1171. 10.1242/jcs.00969 14970264

[GR265595GAOC45] Lee S, Rho SS, Park H, Park JA, Kim J, Lee IK, Koh GY, Mochizuki N, Kim YM, Kwon YG. 2017. Carbohydrate-binding protein CLEC14A regulates VEGFR-2- and VEGFR-3-dependent signals during angiogenesis and lymphangiogenesis. J Clin Invest 127: 457–471. 10.1172/JCI85145 27991863 PMC5272179

[GR265595GAOC46] Leek JT, Storey JD. 2007. Capturing heterogeneity in gene expression studies by surrogate variable analysis. PLoS Genet 3: 1724–1735. 10.1371/journal.pgen.0030161 17907809 PMC1994707

[GR265595GAOC47] Lin C, Ding J, Bar-Joseph Z. 2020. Inferring TF activation order in time series scRNA-Seq studies. PLoS Comput Biol 16: e1007644. 10.1371/journal.pcbi.1007644 32069291 PMC7048296

[GR265595GAOC48] Linderman GC, Rachh M, Hoskins JG, Steinerberger S, Kluger Y. 2019. Fast interpolation-based t-SNE for improved visualization of single-cell RNA-seq data. Nat Methods 16: 243–245. 10.1038/s41592-018-0308-4 30742040 PMC6402590

[GR265595GAOC49] Liu GJ, Cimmino L, Jude JG, Hu Y, Witkowski MT, McKenzie MD, Kartal-Kaess M, Best SA, Tuohey L, Liao Y, 2014. Pax5 loss imposes a reversible differentiation block in B-progenitor acute lymphoblastic leukemia. Genes Dev 28: 1337–1350. 10.1101/gad.240416.114 24939936 PMC4066403

[GR265595GAOC50] Lopes HF, West M. 2004. Bayesian model assessment in factor analysis. Stat Sin 14: 41–67.

[GR265595GAOC51] Lopez R, Regier J, Cole MB, Jordan MI, Yosef N. 2018. Deep generative modeling for single-cell transcriptomics. Nat Methods 15: 1053–1058. 10.1038/s41592-018-0229-2 30504886 PMC6289068

[GR265595GAOC52] MacKay DJC. 1996. Bayesian non-linear modeling for the prediction competition. In Maximum entropy and Bayesian methods (ed. Heidbreder GR), pp. 221–234. Springer, Dordrecht, Netherlands.

[GR265595GAOC53] Miraldi ER, Pokrovskii M, Watters A, Castro DM, De Veaux N, Hall JA, Lee JY, Ciofani M, Madar A, Carriero N, 2019. Leveraging chromatin accessibility for transcriptional regulatory network inference in T helper 17 cells. Genome Res 29: 449–463. 10.1101/gr.238253.118 30696696 PMC6396413

[GR265595GAOC54] Moerman T, Aibar Santos S, Bravo González-Blas C, Simm J, Moreau Y, Aerts J, Aerts S. 2019. GRNBoost2 and Arboreto: efficient and scalable inference of gene regulatory networks. Bioinformatics 35: 2159–2161. 10.1093/bioinformatics/bty916 30445495

[GR265595GAOC55] Moon KR, van Dijk D, Wang Z, Gigante S, Burkhardt DB, Chen WS, Yim K, Elzen AVD, Hirn MJ, Coifman RR, 2019. Visualizing structure and transitions in high-dimensional biological data. Nat Biotechnol 37: 1482–1492. 10.1038/s41587-019-0336-3 31796933 PMC7073148

[GR265595GAOC56] Nguyen TM, Shafi A, Nguyen T, Draghici S. 2019. Identifying significantly impacted pathways: a comprehensive review and assessment. Genome Biol 20: 203. 10.1186/s13059-019-1790-4 31597578 PMC6784345

[GR265595GAOC57] Nutt SL, Heavey B, Rolink AG, Busslinger M. 1999. Commitment to the B-lymphoid lineage depends on the transcription factor Pax5. Nature 401: 556–562. 10.1038/44076 10524622

[GR265595GAOC58] Paul F, Arkin Y, Giladi A, Jaitin DA, Kenigsberg E, Keren-Shaul H, Winter D, Lara-Astiaso D, Gury M, Weiner A, 2016. Transcriptional heterogeneity and lineage commitment in myeloid progenitors. Cell 164: 325. 10.1016/j.cell.2015.12.046 28915372

[GR265595GAOC59] Poncy A, Antoniou A, Cordi S, Pierreux CE, Jacquemin P, Lemaigre FP. 2015. Transcription factors SOX4 and SOX9 cooperatively control development of bile ducts. Dev Biol 404: 136–148. 10.1016/j.ydbio.2015.05.012 26033091

[GR265595GAOC60] R Core Team. 2020. R: a language and environment for statistical computing. R Foundation for Statistical Computing, Vienna. https://www.R-project.org/.

[GR265595GAOC61] Regev A, Teichmann SA, Lander ES, Amit I, Benoist C, Birney E, Bodenmiller B, Campbell P, Carninci P, Clatworthy M, 2017. The Human Cell Atlas. eLife 6: e27041. 10.7554/eLife.27041 29206104 PMC5762154

[GR265595GAOC62] Satija R, Farrell JA, Gennert D, Schier AF, Regev A. 2015. Spatial reconstruction of single-cell gene expression data. Nat Biotechnol 33: 495–502. 10.1038/nbt.3192 25867923 PMC4430369

[GR265595GAOC63] Shapiro E, Biezuner T, Linnarsson S. 2013. Single-cell sequencing-based technologies will revolutionize whole-organism science. Nat Rev Genet 14: 618–630. 10.1038/nrg3542 23897237

[GR265595GAOC64] Specht AT, Li J. 2017. LEAP: constructing gene co-expression networks for single-cell RNA-sequencing data using pseudotime ordering. Bioinformatics 33: 764–766. 10.1093/bioinformatics/btw729 27993778 PMC5860270

[GR265595GAOC65] Suh HC, Gooya J, Renn K, Friedman AD, Johnson PF, Keller JR. 2006. C/EBPα determines hematopoietic cell fate in multipotential progenitor cells by inhibiting erythroid differentiation and inducing myeloid differentiation. Blood 107: 4308–4316. 10.1182/blood-2005-06-2216 16469877 PMC1895788

[GR265595GAOC66] Svensson V, Vento-Tormo R, Teichmann SA. 2018. Exponential scaling of single-cell RNA-seq in the past decade. Nat Protoc 13: 599–604. 10.1038/nprot.2017.149 29494575

[GR265595GAOC67] Tabula Muris Consortium, Overall coordination, Logistical coordination, Organ collection and processing, Library preparation and sequencing, Computational data analysis, Cell type annotation, Writing group, Supplemental text writing group, and Principal investigators. 2018. Single-cell transcriptomics of 20 mouse organs creates a *Tabula Muris*. Nature 562: 367–372. 10.1038/s41586-018-0590-4 30283141 PMC6642641

[GR265595GAOC68] Takahashi S, Komeno T, Suwabe N, Yoh K, Nakajima O, Nishimura S, Kuroha T, Nagasawa T, Yamamoto M. 1998. Role of GATA-1 in proliferation and differentiation of definitive erythroid and megakaryocytic cells in vivo. Blood 92: 434–442. 10.1182/blood.V92.2.434 9657742

[GR265595GAOC69] Treutlein B, Brownfield DG, Wu AR, Neff NF, Mantalas GL, Espinoza FH, Desai TJ, Krasnow MA, Quake SR. 2014. Reconstructing lineage hierarchies of the distal lung epithelium using single-cell RNA-seq. Nature 509: 371–375. 10.1038/nature13173 24739965 PMC4145853

[GR265595GAOC70] Wainwright MJ, Jordan MI. 2008. Graphical models, exponential families, and variational inference. Foundations and Trends in Machine Learning, Vol. 1, pp. 1–305. Now Publishers, Inc., Boston. 10.1561/2200000001

[GR265595GAOC71] Wang D, Gu J. 2018. VASC: dimension reduction and visualization of single-cell RNA-seq data by deep variational autoencoder. Genom Proteom Bioinf 16: 320–331. 10.1016/j.gpb.2018.08.003 PMC6364131 30576740

[GR265595GAOC72] Wang B, Ramazzotti D, De Sano L, Zhu J, Pierson E, Batzoglou S. 2018. SIMLR: a tool for large-scale genomic analyses by multi-kernel learning. Proteomics 18: 1700232. 10.1002/pmic.201700232 29265724

[GR265595GAOC73] Wu X, Briseño CG, Durai V, Albring JC, Haldar M, Bagadia P, Kim KW, Randolph GJ, Murphy TL, Murphy KM. 2016. *Mafb* lineage tracing to distinguish macrophages from other immune lineages reveals dual identity of Langerhans cells. J Exp Med 213: 2553–2565. 10.1084/jem.20160600 27810926 PMC5110021

[GR265595GAOC74] Wu Y, Tamayo P, Zhang K. 2018. Visualizing and interpreting single-cell gene expression datasets with similarity weighted nonnegative embedding. Cell Syst 7: 656–666.e4. 10.1016/j.cels.2018.10.015 30528274 PMC6311449

[GR265595GAOC75] Yevshin I, Sharipov R, Kolmykov S, Kondrakhin Y, Kolpakov F. 2019. GTRD: a database on gene transcription regulation—2019 update. Nucleic Acids Res 47: D100–D105. 10.1093/nar/gky1128 30445619 PMC6323985

[GR265595GAOC76] Yuan Y, Bar-Joseph Z. 2019. Deep learning for inferring gene relationships from single-cell expression data. Proc Natl Acad Sci 116: 27151–27158. 10.1073/pnas.1911536116 PMC6936704 31822622

[GR265595GAOC77] Zappia L, Phipson B, Oshlack A. 2018. Exploring the single-cell RNA-seq analysis landscape with the scRNA-tools database. PLoS Comput Biol 14: e1006245. 10.1371/journal.pcbi.1006245 29939984 PMC6034903

[GR265595GAOC78] Zeisel A, Munoz-Manchado AB, Codeluppi S, Lonnerberg P, La Manno G, Jureus A, Marques S, Munguba H, He L, Betsholtz C, 2015. Brain structure. Cell types in the mouse cortex and hippocampus revealed by single-cell RNA-seq. Science 347: 1138–1142. 10.1126/science.aaa1934 25700174

[GR265595GAOC79] Zhao BS, Roundtree IA, He C. 2018. Publisher correction: post-transcriptional gene regulation by mRNA modifications. Nat Rev Mol Cell Biol 19: 808. 10.1038/s41580-018-0075-1 30341428

[GR265595GAOC80] Zheng GX, Terry JM, Belgrader P, Ryvkin P, Bent ZW, Wilson R, Ziraldo SB, Wheeler TD, McDermott GP, Zhu J, 2017. Massively parallel digital transcriptional profiling of single cells. Nat Commun 8: 14049. 10.1038/ncomms14049 28091601 PMC5241818

[GR265595GAOC81] Žurauskienė J, Yau C. 2016. pcaReduce: hierarchical clustering of single cell transcriptional profiles. BMC Bioinformatics 17: 140. 10.1186/s12859-016-0984-y 27005807 PMC4802652

