# Supplementary material for: A Bayesian inference transcription factor activity model for the analysis of single-cell transcriptomes
Source: Genome Res. 2021 Jul;31(7):1296–311. doi: 10.1101/gr.265595.120 (PMC8256867; doi:10.1101/gr.265595.120)
Supplement: Supplemental Material [file supp_31_7_1296__DC1.html]

A Bayesian inference transcription factor activity model for the analysis of single-cell transcriptomes — A Bayesian inference transcription factor activity model for the analysis of single-cell transcriptomes — Supplemental Material 

# A Bayesian inference transcription factor activity model for the analysis of single-cell transcriptomes

## Supplemental Material

- Supplemental\_Code.zip
- Supplemental\_Fig\_S1.pdf
- Supplemental\_Fig\_S2.pdf
- Supplemental\_Fig\_S3.pdf
- Supplemental\_Fig\_S4.pdf
- Supplemental\_Fig\_S5.pdf
- Supplemental\_Fig\_S6.pdf
- Supplemental\_Fig\_S7.pdf
- Supplemental\_Fig\_S8.pdf
- Supplemental\_Fig\_S9.pdf
- Supplemental\_Fig\_S10.pdf
- Supplemental\_Fig\_S11.pdf
- Supplemental\_Fig\_S12.pdf
- Supplemental\_Fig\_S13.pdf
- Supplemental\_Fig\_S14.pdf
- Supplemental\_Fig\_S15.pdf
- Supplemental\_Fig\_S16.pdf
- Supplemental\_Fig\_S17.pdf
- Supplemental\_Fig\_S18.pdf
- Supplemental\_Fig\_S19.pdf
- Supplemental\_Fig\_S20.pdf
- Supplemental\_Fig\_S21.pdf
- Supplemental\_Table\_S1.xlsx
