# Supplementary material for: A Bayesian inference transcription factor activity model for the analysis of single-cell transcriptomes
Source: Genome Res. 2021 Jul;31(7):1296–311. doi: 10.1101/gr.265595.120 (PMC8256867; doi:10.1101/gr.265595.120)
Supplement: Supplemental Material [file supp_gr.265595.120_Supplemental_Fig_S1.pdf]

	Tabula Muris lung	*Tabula Muris* heart	*Tabula Muris* brain	Blood cell development	CRISPRi
Number of cells	5449	4365	3401	2730	5174
Number of most variable expressed genes	4552	5337	5617	1830	4844
Number of transcription factors	106	87	105	106	103

Figure S1: The description of the datasets used in this manuscript.

Detailed statistics for the number of cells, variably expressed genes and transcription factor targets for the datasets analyzed.

Fig.S1
