# Supplementary material for: A Bayesian inference transcription factor activity model for the analysis of single-cell transcriptomes
Source: Genome Res. 2021 Jul;31(7):1296–311. doi: 10.1101/gr.265595.120 (PMC8256867; doi:10.1101/gr.265595.120)
Supplement: Supplemental Material [file supp_gr.265595.120_Supplemental_Fig_S3.pdf]

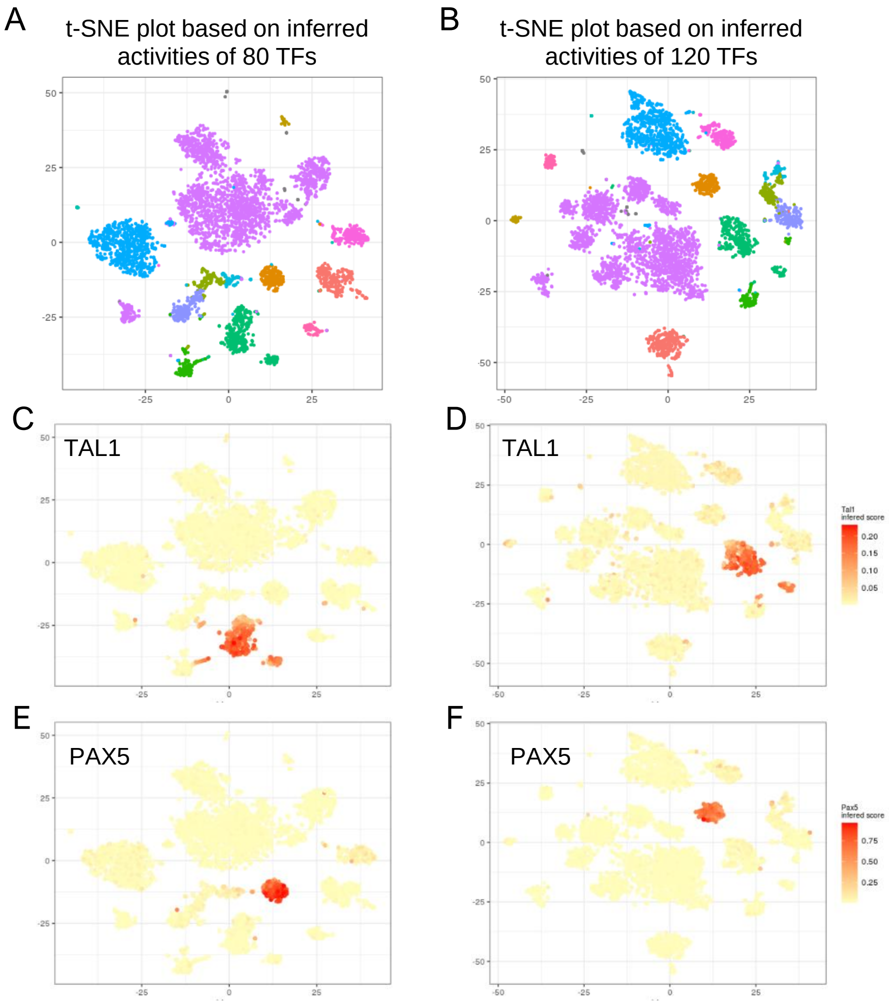

Figure. S3: Model robustness with distinct numbers of TFs in the *Tabula Muris* lung dataset:

A, t-SNE plots of BITFAM inferred activities of either 80 TFs (**A**) or 120 TFs (**B**) and examples of corresponding inferred TF activities of the TFs TAL1 (**C, D**) and PAX5 (**E, F**) using 80 TFs or 120 TFs. To evaluate the robustness our model, we changed the number of transcription factors by selecting the top variably expressed TFs, using either 80 or 120 TFs. Then we generated t-SNE plots on the inferred activity matrix derived from the analyses with either 80 or 120 TFs.

Fig.S3
