# Supplementary material for: A Bayesian inference transcription factor activity model for the analysis of single-cell transcriptomes
Source: Genome Res. 2021 Jul;31(7):1296–311. doi: 10.1101/gr.265595.120 (PMC8256867; doi:10.1101/gr.265595.120)
Supplement: Supplemental Material [file supp_gr.265595.120_Supplemental_Fig_S4.pdf]

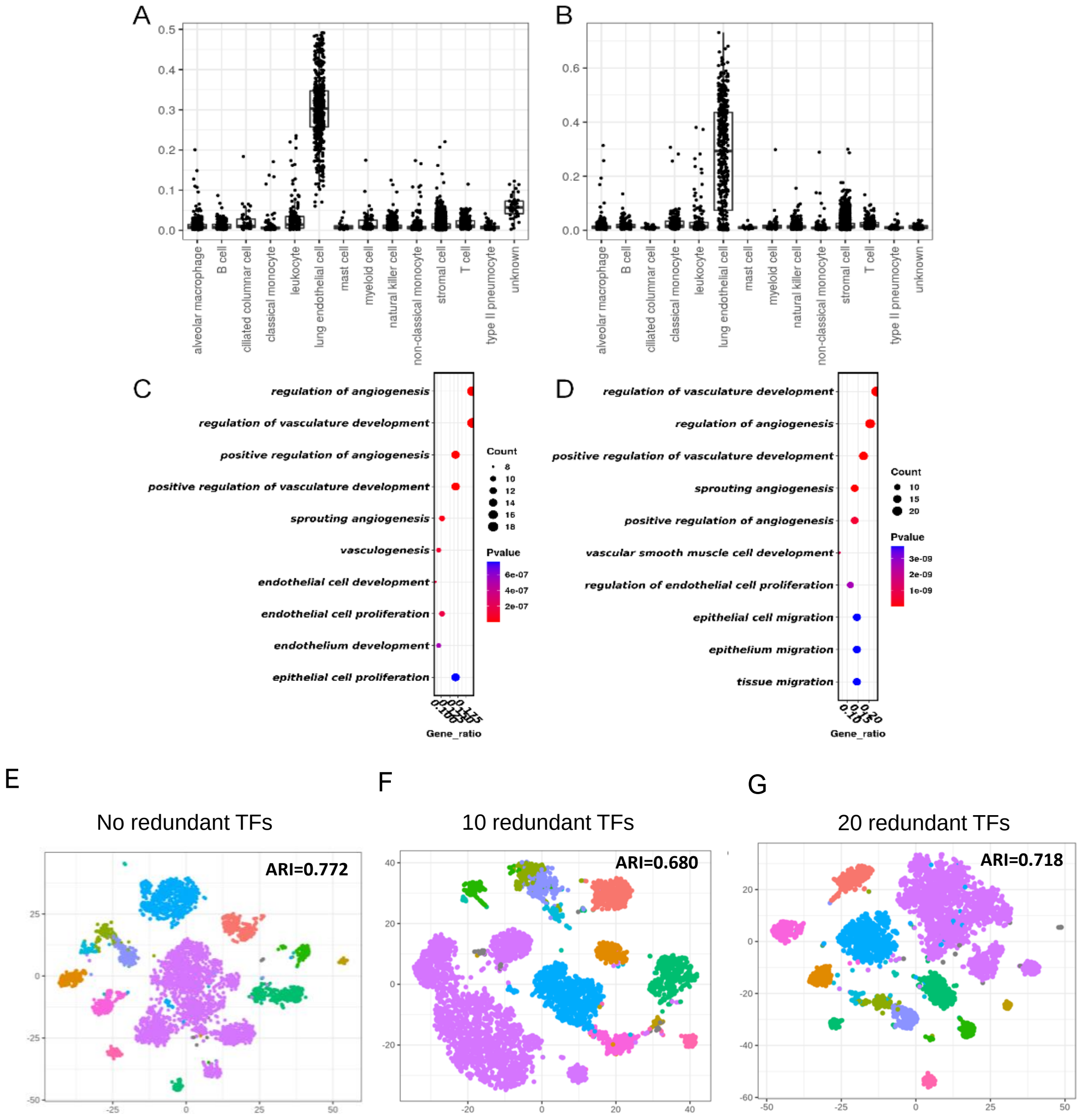


**Figure. S4: The model robustness with redundant TFs in the *Tabula Muris* lung dataset:**

**A**,**B**, The inferred activities of TAL1 in all cell types in the *Tabula Muris* lung dataset. **C,D**, Top 10 significant GO terms of TAL1 top weighted target genes in the *Tabula Muris* lung dataset. t-SNE plots of BITFAM inferred TF activities with colors reflecting the biological cell type identify (defined by the *Tabula Muris*) without redundant TFs (**E**), with 10 redundant TFs (**F**) or with 20 redundant TFs (**G**). In order to assess the possible impact of redundant TFs, we added either 10 or 20 redundant transcription factors with their ChIP-seq targets when applying BITFAM to the *Tabula Muris* lung data. We removed the newly added redundant transcription factors from the Z matrix and generated t-SNE plots of the TF inferred activities. The results show that cells of the same color (indicating a biological cell identity) continue to cluster together regardless of the presence of redundant TFs. The ARIs of the clustering are decreased in when redundant TFs are included.

**Fig.S4**
