# Supplementary material for: A Bayesian inference transcription factor activity model for the analysis of single-cell transcriptomes
Source: Genome Res. 2021 Jul;31(7):1296–311. doi: 10.1101/gr.265595.120 (PMC8256867; doi:10.1101/gr.265595.120)
Supplement: Supplemental Material [file supp_gr.265595.120_Supplemental_Fig_S5.pdf]

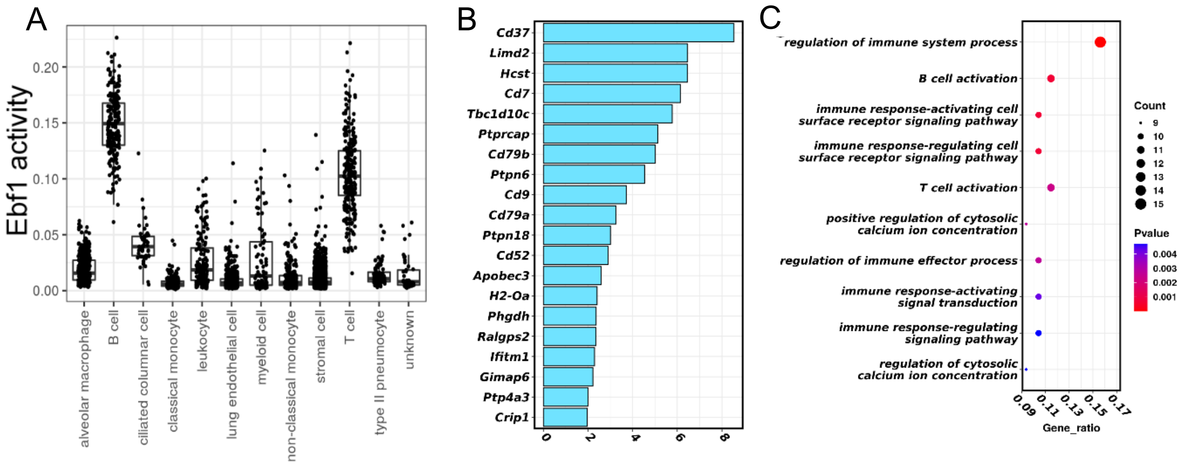

Figure S5: BITFAM results for the TF EBF1 in the *Tabula Muris* lung dataset

A, Boxplot of inferred activities of the TF EBF1 in each cell type in the *Tabula Muris* lung data. **B**, Top weighted genes of EBF1. **C**, Top 10 significant GO terms of EBF1 enriched by the top 100 positively weighted genes learnt from the *Tabula Muris* lung data.

Fig.S5
