# Supplementary material for: A Bayesian inference transcription factor activity model for the analysis of single-cell transcriptomes
Source: Genome Res. 2021 Jul;31(7):1296–311. doi: 10.1101/gr.265595.120 (PMC8256867; doi:10.1101/gr.265595.120)
Supplement: Supplemental Material [file supp_gr.265595.120_Supplemental_Fig_S6.pdf]

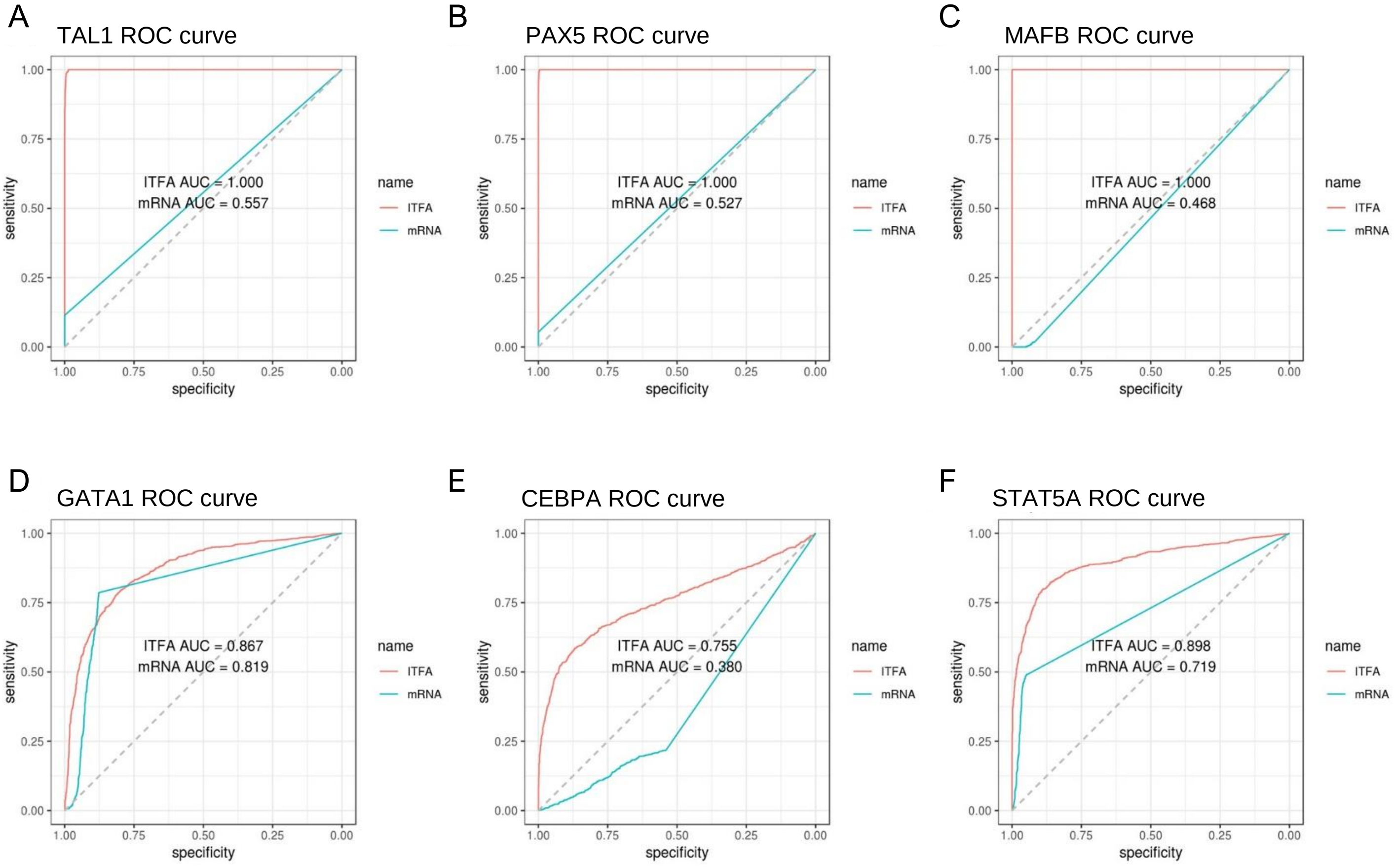

Figure S6: Receiver operating characteristic (ROC) curve for mRNA levels and inferred TF activity (ITFA) for the identification of cell types.

A, ROC curve for *Tal1* mRNA levels and inferred activity for identifying endothelial cells in the *Tabula Muris* lung data. **B**, ROC curve for *Pax5* mRNA levels and inferred activity for identifying B cells in the *Tabula Muris* lung data. **C**, ROC curve for *Mafb* mRNA levels and inferred activity for identifying alveolar macrophages in the *Tabula Muris* lung data. **D**, ROC curve for *Gata1* mRNA levels and inferred activity for identifying Megakaryocyte-erythroid progenitors (MEP) in the blood cell development data. **E**. ROC curve for *Cebpa* mRNA levels and inferred activity for identifying Granulocyte-macrophage progenitors (GMP) in the blood cell development data. **F**, ROC curve for *Stat5a* mRNA levels and inferred activity for identifying Granulocyte-macrophage progenitors (GMP) in the blood cell development data.

Fig.S6
