# Supplementary material for: A Bayesian inference transcription factor activity model for the analysis of single-cell transcriptomes
Source: Genome Res. 2021 Jul;31(7):1296–311. doi: 10.1101/gr.265595.120 (PMC8256867; doi:10.1101/gr.265595.120)
Supplement: Supplemental Material [file supp_gr.265595.120_Supplemental_Fig_S7.pdf]

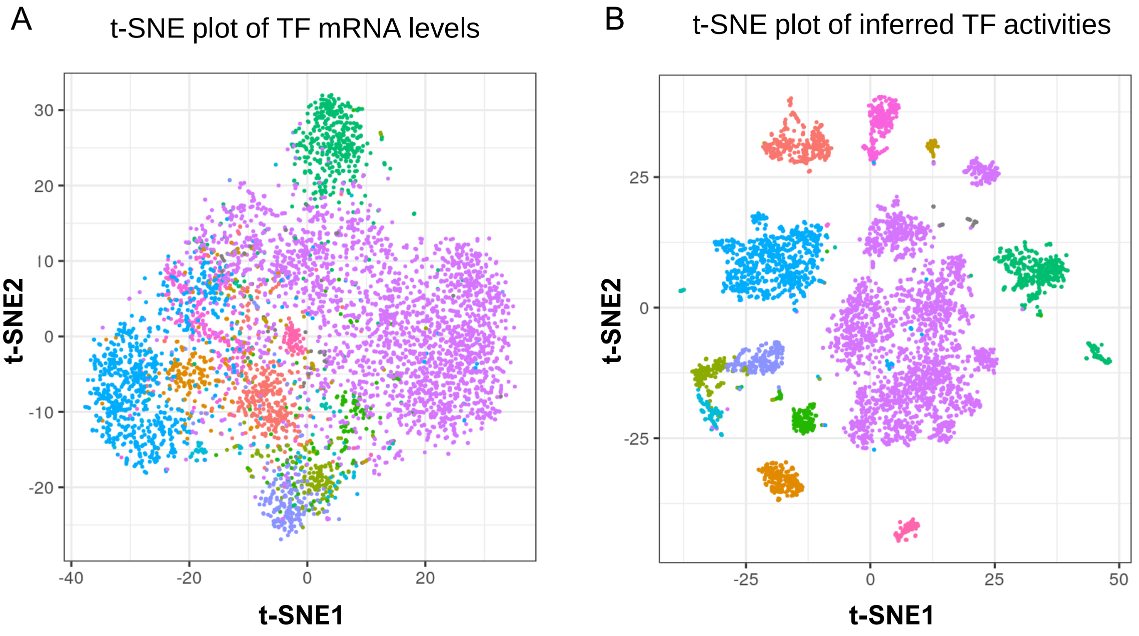

Figure. S7: The comparison of TF mRNA expression levels and inferred activities

tSNE plot of the *Tabula Muris* lung dataset using mRNA expression levels of TFs (**A**) or BITFAM inferred TF activities (**B**).

Fig.S7
