# Supplementary material for: A Bayesian inference transcription factor activity model for the analysis of single-cell transcriptomes
Source: Genome Res. 2021 Jul;31(7):1296–311. doi: 10.1101/gr.265595.120 (PMC8256867; doi:10.1101/gr.265595.120)
Supplement: Supplemental Material [file supp_gr.265595.120_Supplemental_Fig_S8.pdf]

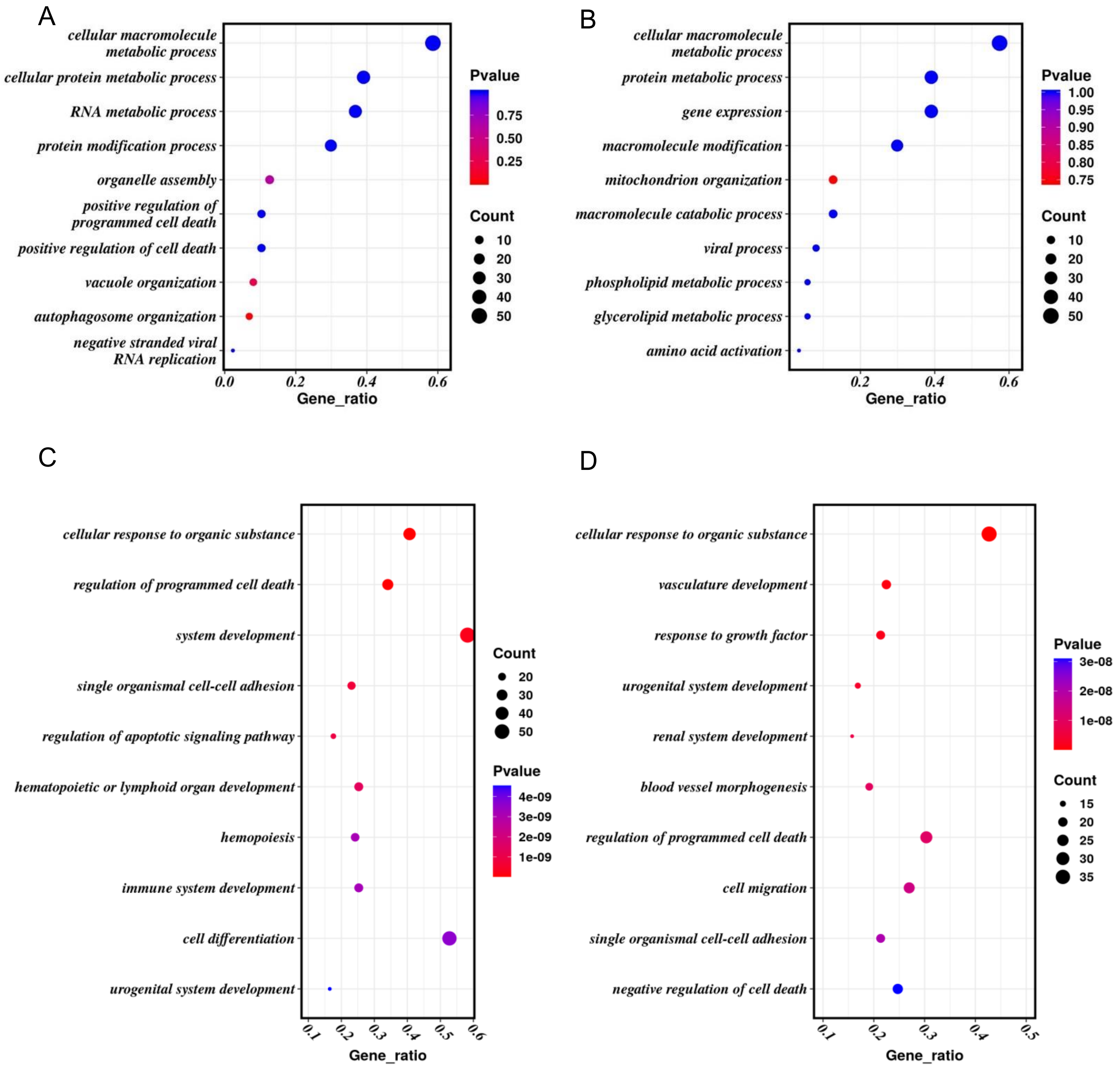

Figure S8: Enrichment analysis of ChIP-seq target genes of TAL1 and Pax5

A, Top 10 significant GO terms of randomly sampled 100 TAL1 ChIP-seq target genes in the *Tabula Muris* lung dataset. **B**, Top 10 significant GO terms of randomly sampled 100 PAX5 ChIP-seq target genes. **C**, Top 10 significant GO terms of 100 most expressed TAL1 ChIP-seq target genes. **D**, Top 10 significant GO terms of the 100 most expressed PAX5 ChIP-seq target genes.

Fig.S8
