# Supplementary material for: A Bayesian inference transcription factor activity model for the analysis of single-cell transcriptomes
Source: Genome Res. 2021 Jul;31(7):1296–311. doi: 10.1101/gr.265595.120 (PMC8256867; doi:10.1101/gr.265595.120)
Supplement: Supplemental Material [file supp_gr.265595.120_Supplemental_Fig_S9.pdf]

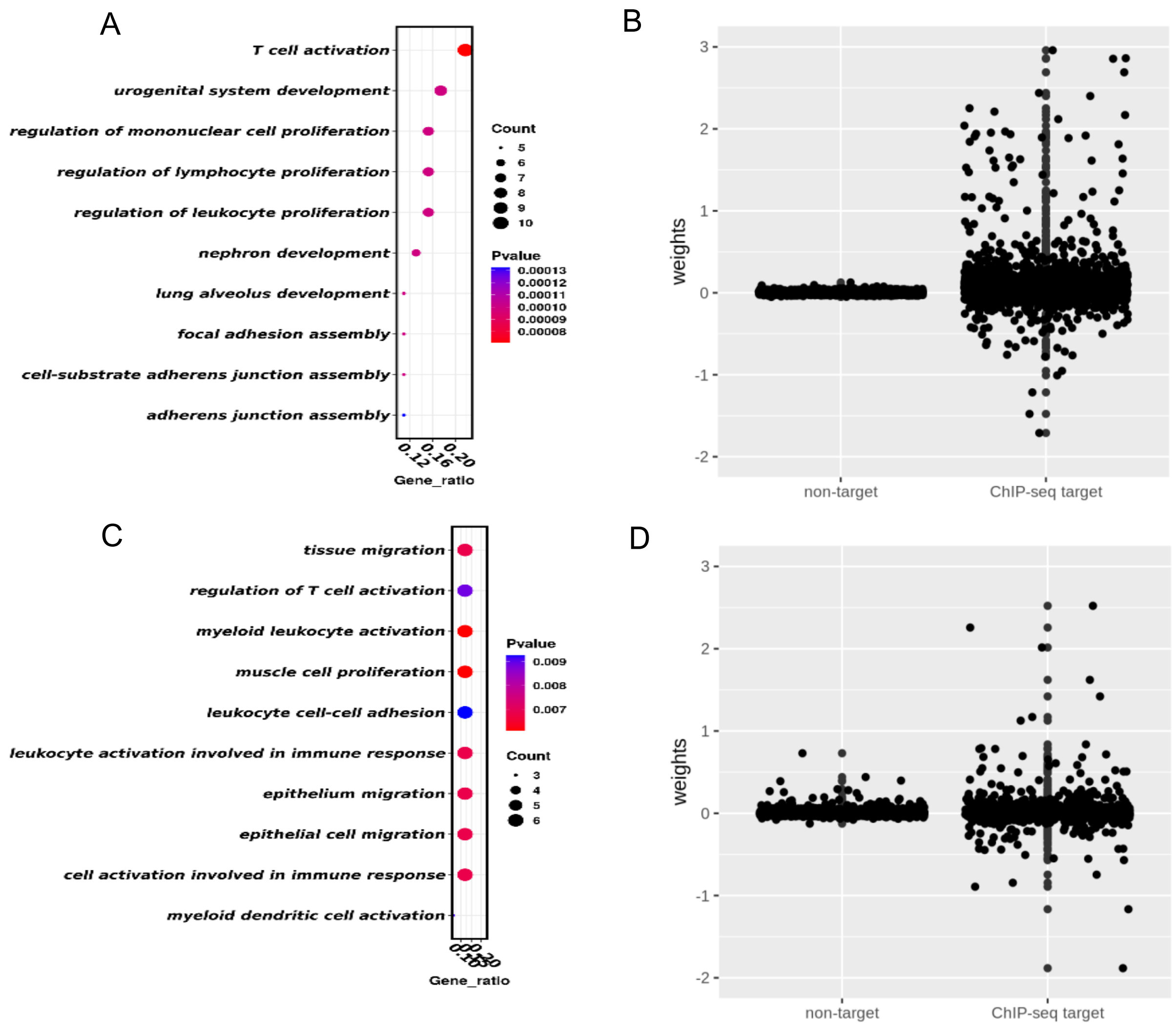

Figure S9: Genes with negative weights derived from the Z-Matrix following BITFAM inferences of PAX5 and MAFB TF activities.

A, Gene signatures of negatively weighted genes for PAX5**. B,** Boxplot of BITFAM inferred weights for PAX5 grouped by whether they are ChIP-seq target genes on non-target genes. **C,** Gene signatures of negatively weighted genes for MAFB. **D,** Boxplot of BITFAM inferred weights for MAFB grouped by whether they are ChIP-seq target genes on non-target genes.

Fig.S9
