# Supplementary material for: A Bayesian inference transcription factor activity model for the analysis of single-cell transcriptomes
Source: Genome Res. 2021 Jul;31(7):1296–311. doi: 10.1101/gr.265595.120 (PMC8256867; doi:10.1101/gr.265595.120)
Supplement: Supplemental Material [file supp_gr.265595.120_Supplemental_Fig_S10.pdf]

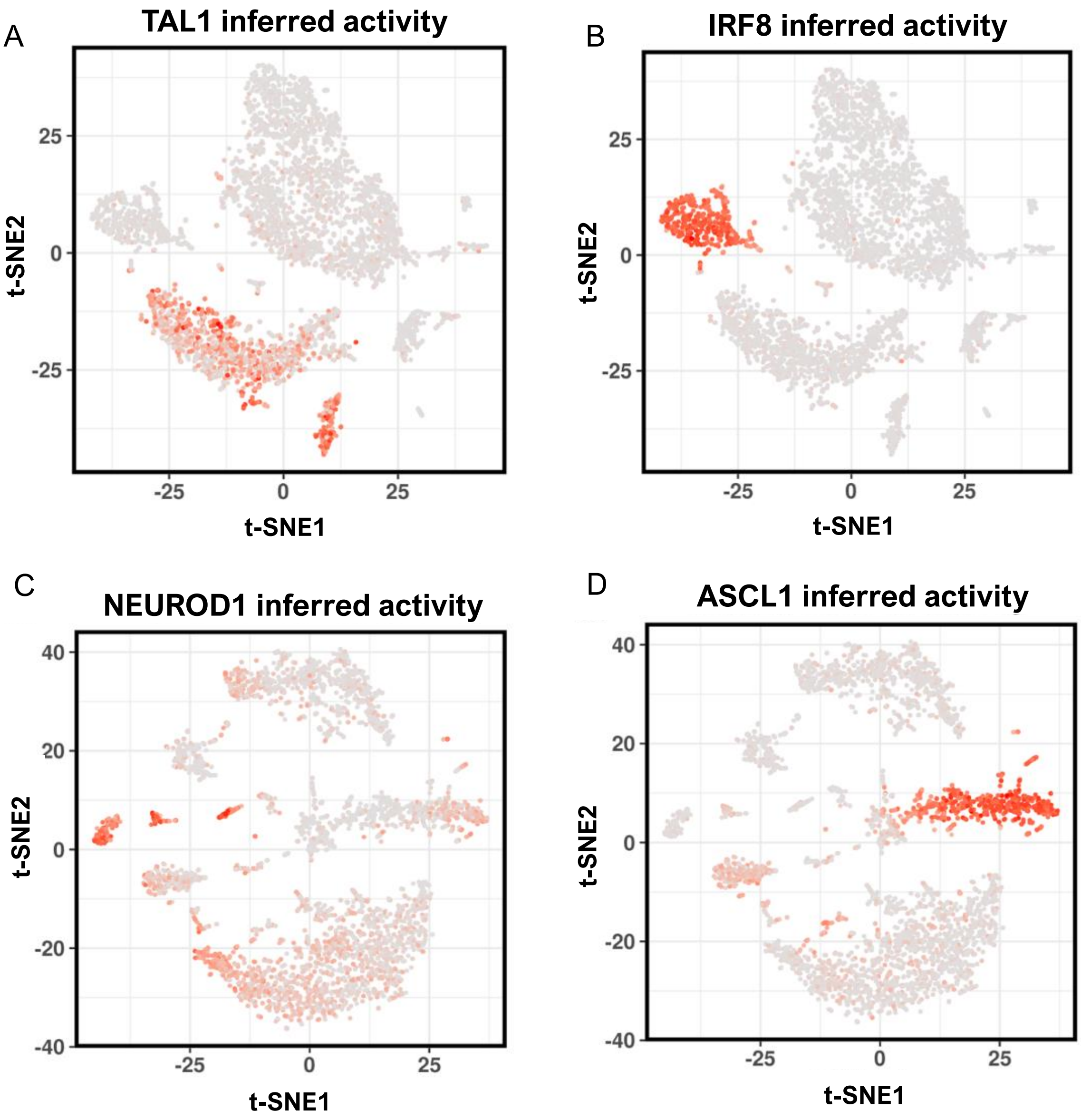

Figure S10: Transcription factors activities inferred by BITFAM in the *Tabula Muris* heart and brain data sets.

The inferred activities of the transcription factors TAL1 **(A)** and IRF8 **(B)** in the *Tabula Muris* heart dataset and of NEUROD1 (**C**) and ASCL1 (**D**) in the *Tabula Muris* brain dataset.

Fig.S10
