# Supplementary material for: A Bayesian inference transcription factor activity model for the analysis of single-cell transcriptomes
Source: Genome Res. 2021 Jul;31(7):1296–311. doi: 10.1101/gr.265595.120 (PMC8256867; doi:10.1101/gr.265595.120)
Supplement: Supplemental Material [file supp_gr.265595.120_Supplemental_Fig_S11.pdf]

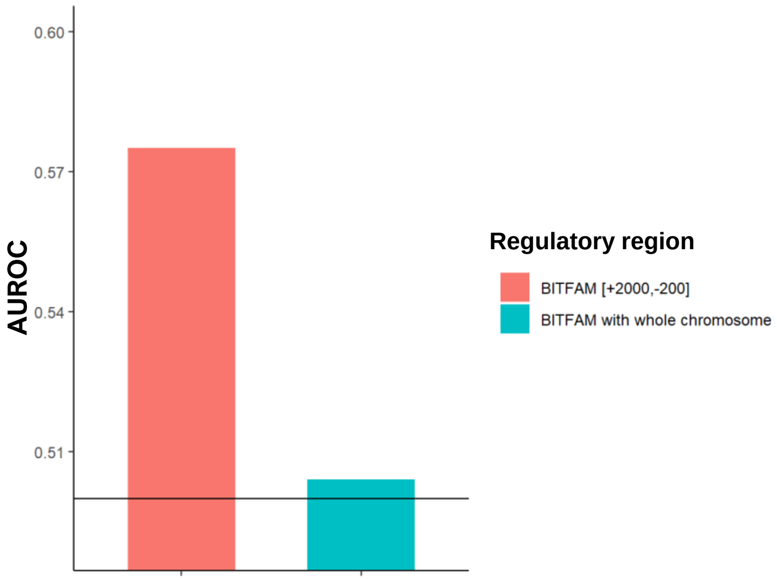

Figure S11. BITFAM performance using distal ChIP-seq signals

We compared the BITFAM AUROC using the focused [-2000, +200] region to identify ChIP-seq targets or to extend the potential regulatory regions to the whole chromosome and found that the extending the region to include all potential regulatory regions on the chromosome reduces its performance.

Fig.S11
