# Supplementary material for: A Bayesian inference transcription factor activity model for the analysis of single-cell transcriptomes
Source: Genome Res. 2021 Jul;31(7):1296–311. doi: 10.1101/gr.265595.120 (PMC8256867; doi:10.1101/gr.265595.120)
Supplement: Supplemental Material [file supp_gr.265595.120_Supplemental_Fig_S12.pdf]

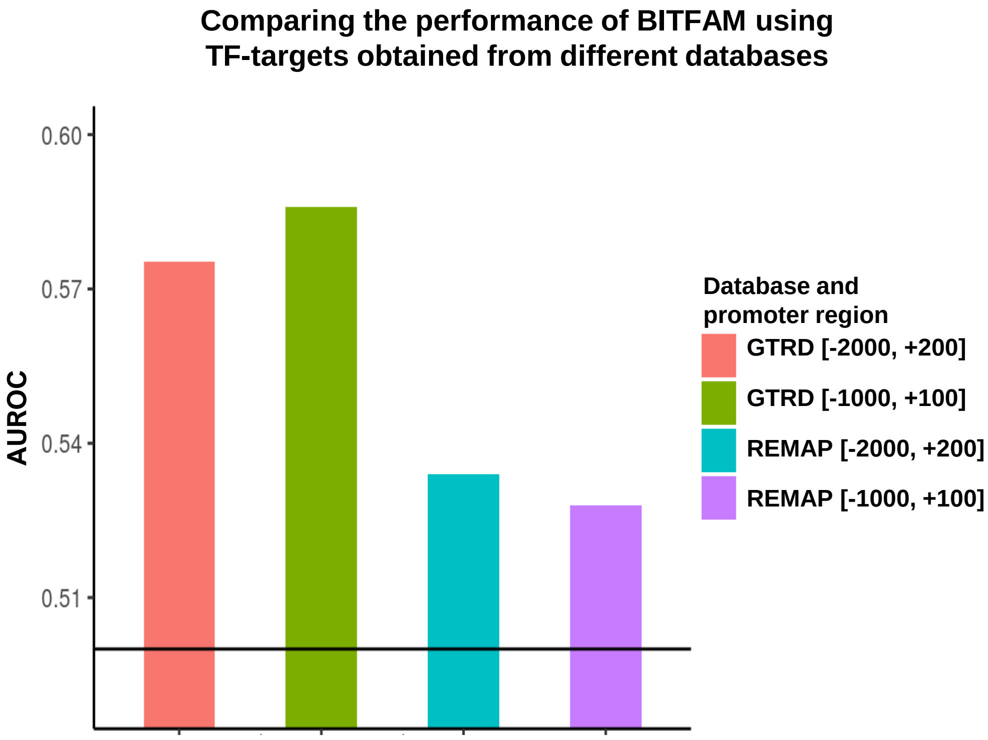

Figure S12. Comparison of distinct ChIP-seq input databases

Comparison of the performance of BITFAM using the default Gene Transcription Regulation Database (GTRD) ChIP-seq database versus the REMAP database. When applying BITFAM to the CRISPRi dataset, we found that using GTRD performed better than when using ReMAP (AUC 0.581 for GTRD on region [-1000, +100], AUC 0.575 for GTRD on region [-2000, +200], AUC 0.552 for ReMAP on region [-1000, +100], AUC 0.547 for ReMap on region [-2000, +200]).

Fig.S12
