# Supplementary material for: A Bayesian inference transcription factor activity model for the analysis of single-cell transcriptomes
Source: Genome Res. 2021 Jul;31(7):1296–311. doi: 10.1101/gr.265595.120 (PMC8256867; doi:10.1101/gr.265595.120)
Supplement: Supplemental Material [file supp_gr.265595.120_Supplemental_Fig_S13.pdf]

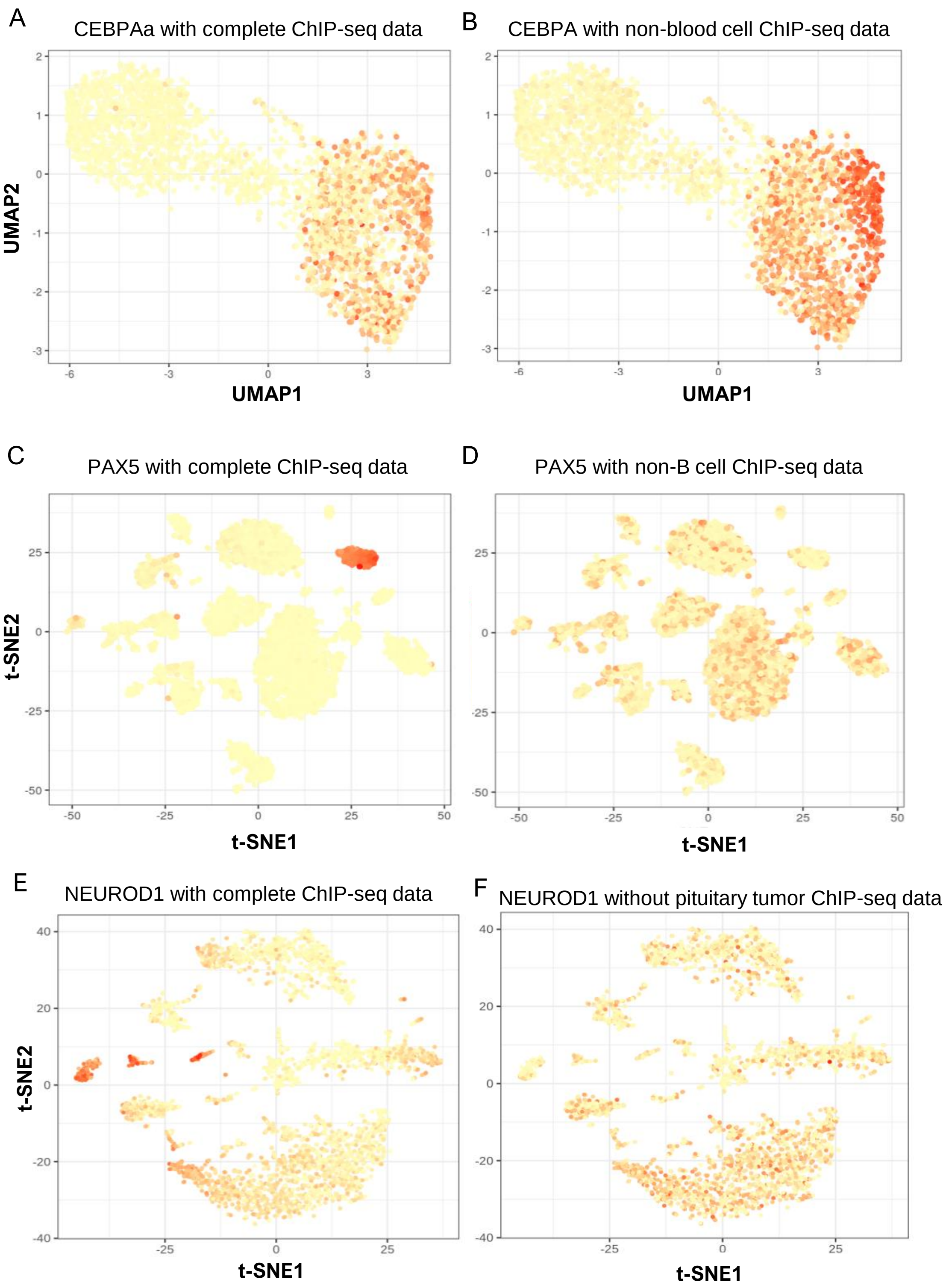

Figure. S13: The inferred activities before and after partially removing ChIP-seq datasets

Inferred CEBPA activities in the blood cell development dataset using all available ChIP-seq data **(A)** or only ChIP-seq data derived from non-blood cells (**B). I**nferred PAX5 activities in the *Tabula Muris* lung dataset using all available ChIP-seq data **(C)** or only ChIP-seq data derived from non-B cells (**D). I**nferred NEUROD1 activities in the *Tabula Muris* Brain dataset using all ChIP-seq data **(E)** or only ChIP-seq data without pituitary tumor ChIP-seq results **(F)**.

Fig.S13
