# Supplementary material for: A Bayesian inference transcription factor activity model for the analysis of single-cell transcriptomes
Source: Genome Res. 2021 Jul;31(7):1296–311. doi: 10.1101/gr.265595.120 (PMC8256867; doi:10.1101/gr.265595.120)
Supplement: Supplemental Material [file supp_gr.265595.120_Supplemental_Fig_S14.pdf]

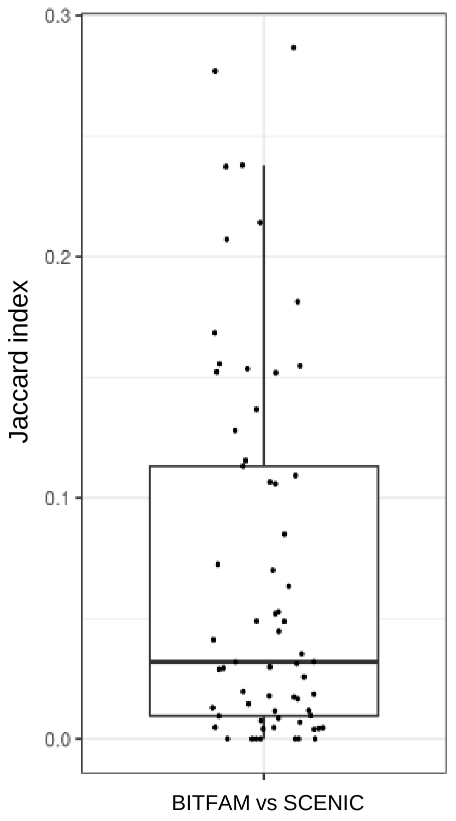

Figure S14: The overlap of target genes identified by BITFAM and SCENIC

Boxplot of the Jaccard index to quantify the overlap between target genes identified by SCENIC and BITFAM.

Fig.S14
