# Supplementary material for: A Bayesian inference transcription factor activity model for the analysis of single-cell transcriptomes
Source: Genome Res. 2021 Jul;31(7):1296–311. doi: 10.1101/gr.265595.120 (PMC8256867; doi:10.1101/gr.265595.120)
Supplement: Supplemental Material [file supp_gr.265595.120_Supplemental_Fig_S15.pdf]

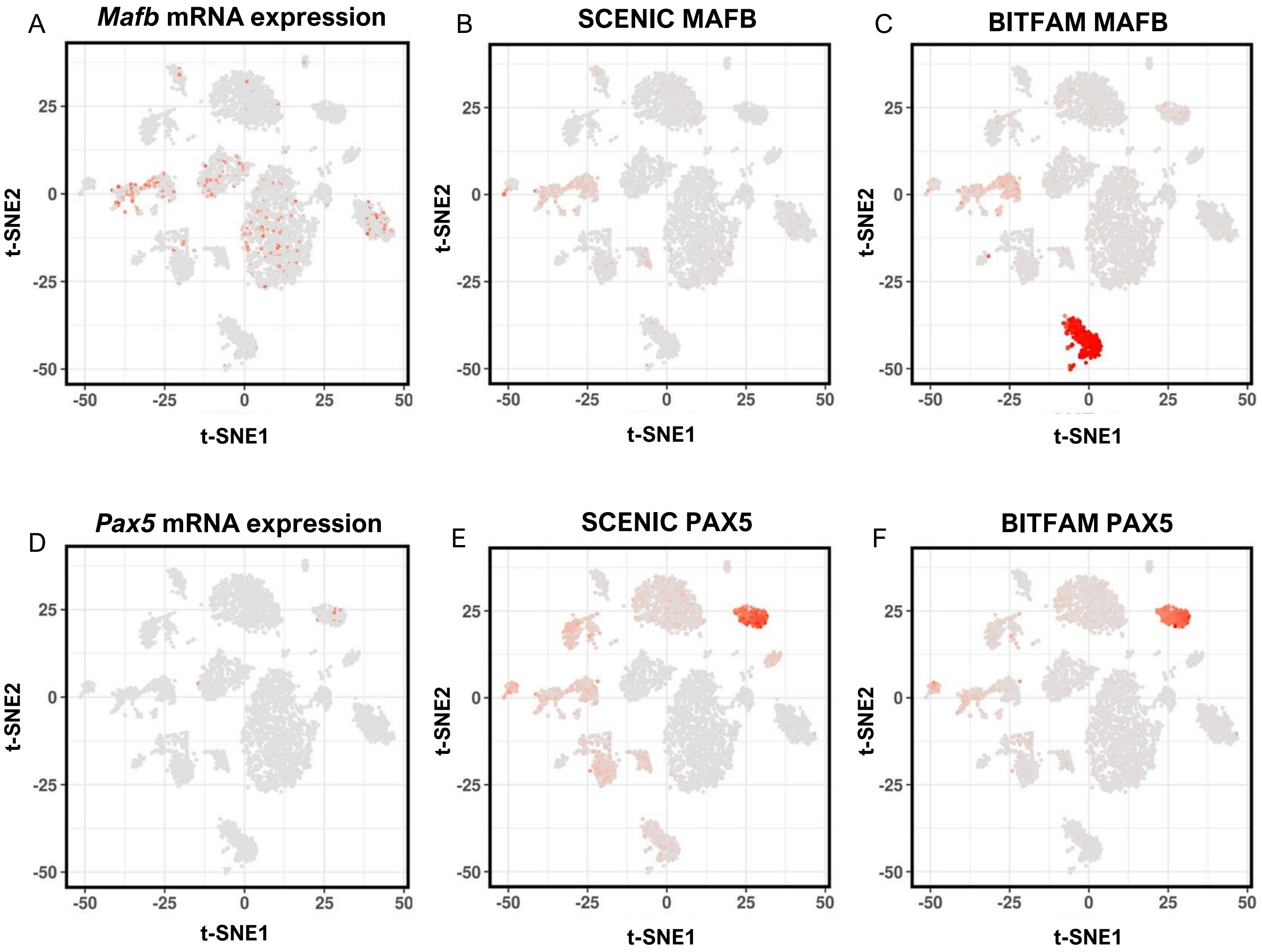


**Figure S15: The activities of MAFB and PAX5 inferred by SCENIC and BITFAM**

**A**, Log-normalized mRNA expression of *Mafb* in the *Tabula Muris* lung data, re-plotted from Fig.2a. **B**, MAFB activity inferred by SCENIC. **C**, MAFB activity inferred by BITFAM. **D**, log-normalized mRNA expression of *Pax5*. **E**, Pax5 activity inferred by SCENIC**.** **F**, PAX5 activity inferred by BITFAM.

**Fig.S15**
