# Supplementary material for: A Bayesian inference transcription factor activity model for the analysis of single-cell transcriptomes
Source: Genome Res. 2021 Jul;31(7):1296–311. doi: 10.1101/gr.265595.120 (PMC8256867; doi:10.1101/gr.265595.120)
Supplement: Supplemental Material [file supp_gr.265595.120_Supplemental_Fig_S16.pdf]

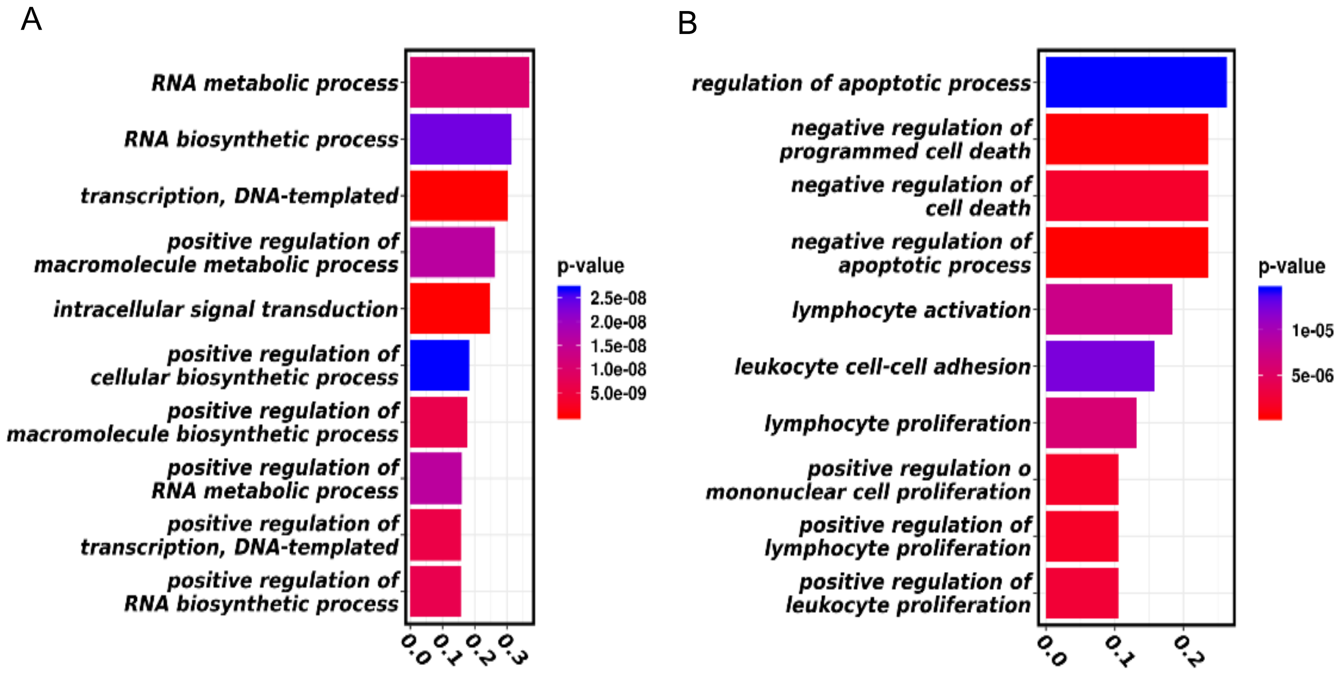

Figure S16: Enrichment analysis of SCENIC target genes and BITFAM target genes for the transcription factor REL-B

A, Top 10 significant GO terms of inferred SCENIC target genes of REL-B in the *Tabula Muris* lung data. **B,** Top 10 significant GO terms of the top 100 inferred BITFAM target genes of REL-B in the *Tabula Muris* lung dataset.

Fig.S16
