# Supplementary material for: A Bayesian inference transcription factor activity model for the analysis of single-cell transcriptomes
Source: Genome Res. 2021 Jul;31(7):1296–311. doi: 10.1101/gr.265595.120 (PMC8256867; doi:10.1101/gr.265595.120)
Supplement: Supplemental Material [file supp_gr.265595.120_Supplemental_Fig_S18.pdf]

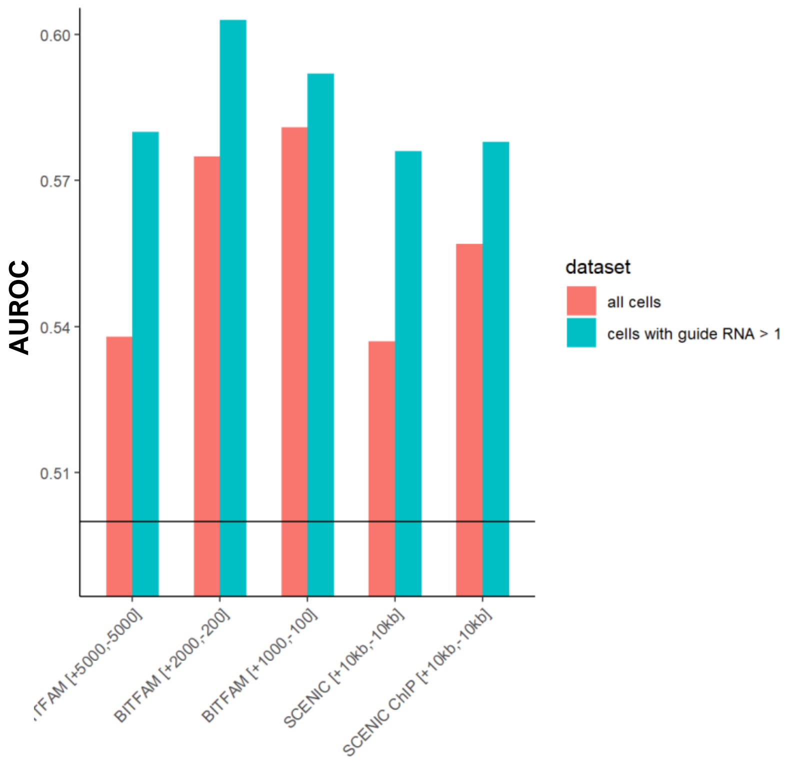

Figure S18: The performance of BITFAM and SCENIC (AUROC) applied to the CRISPRi dataset with and without filtering for cells in which more than one guide RNA was detected by single cell RNA-sequencing.

Fig.S18
