# Supplementary material for: A Bayesian inference transcription factor activity model for the analysis of single-cell transcriptomes
Source: Genome Res. 2021 Jul;31(7):1296–311. doi: 10.1101/gr.265595.120 (PMC8256867; doi:10.1101/gr.265595.120)
Supplement: Supplemental Material [file supp_gr.265595.120_Supplemental_Fig_S19.pdf]

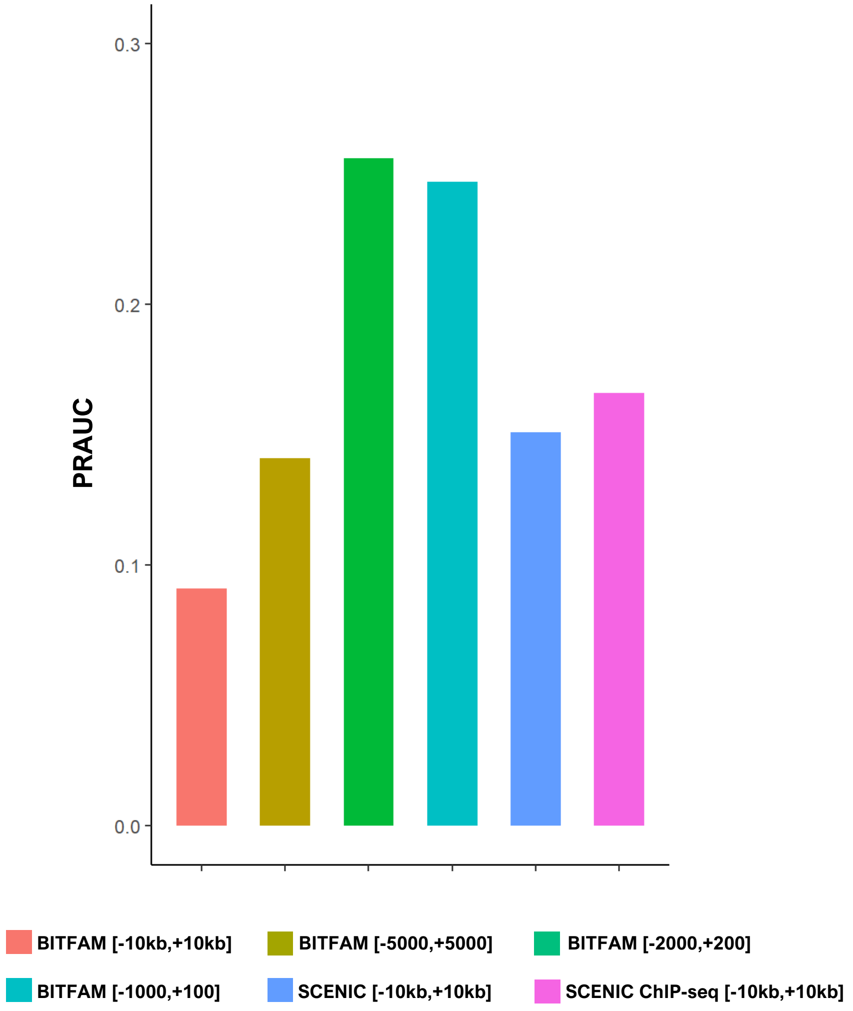

Figure S19: The Precision-Recall AUC (PRAUC) of BITFAM and SCENIC applied on the CRISPRi dataset with 8 TFs for which TF activities were inferred by all methods

Fig.S19
