# Supplementary material for: A Bayesian inference transcription factor activity model for the analysis of single-cell transcriptomes
Source: Genome Res. 2021 Jul;31(7):1296–311. doi: 10.1101/gr.265595.120 (PMC8256867; doi:10.1101/gr.265595.120)
Supplement: Supplemental Material [file supp_gr.265595.120_Supplemental_Fig_S20.pdf]

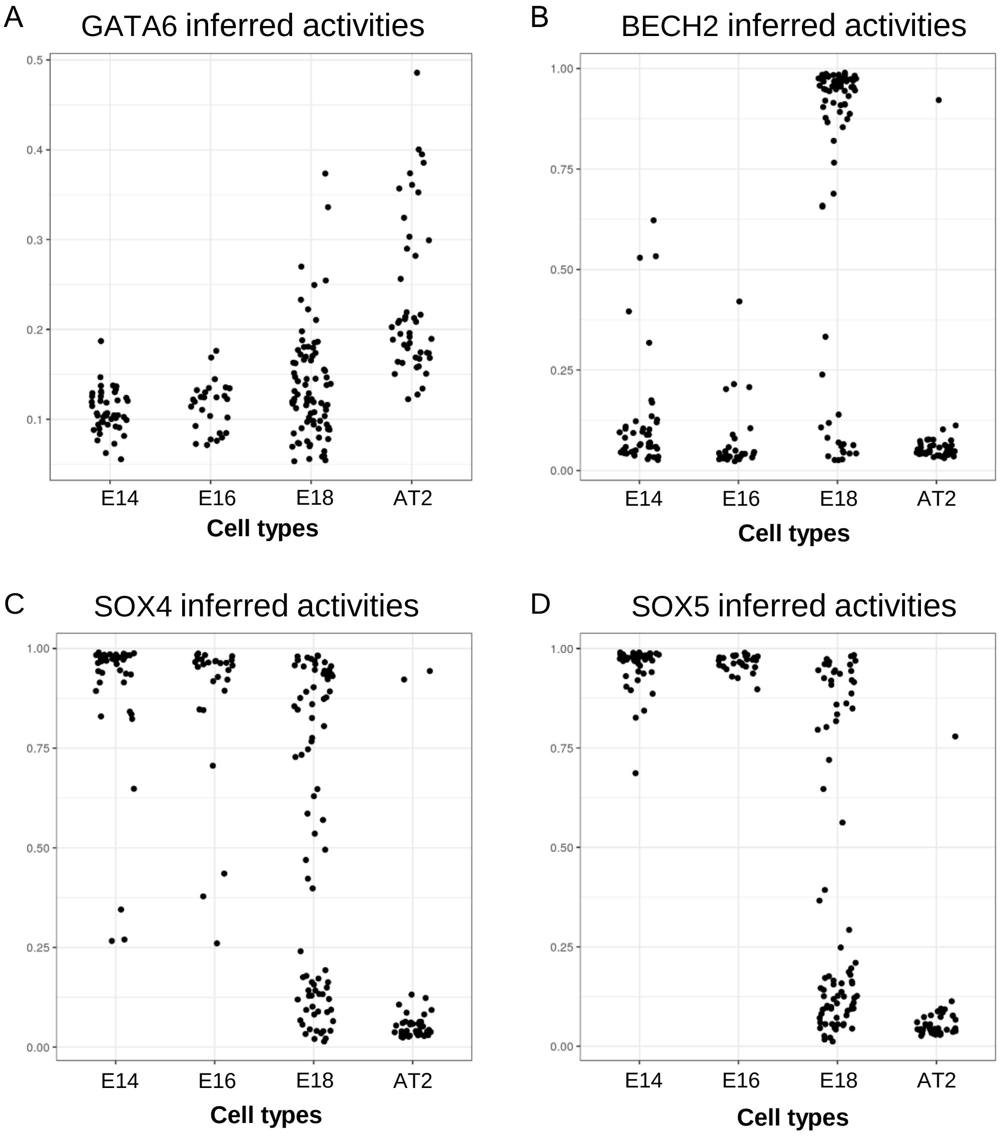

Figure. S20: BITFAM inferred TF activities in a lung development dataset

A, The inferred activities of GATA6. **B**, The inferred activities of BECH2. **C,** The inferred activities of SOX4. **D**, The inferred activities of SOX5. We used BITFAM in a lung development dataset with 152 cells, 15K genes and 3 time points from Lin et al, PLoS Computational Biology (2020), where the results of applying CSHMM-TF to this dataset are provided.

Fig.S20
