# Supplementary material for: A Bayesian inference transcription factor activity model for the analysis of single-cell transcriptomes
Source: Genome Res. 2021 Jul;31(7):1296–311. doi: 10.1101/gr.265595.120 (PMC8256867; doi:10.1101/gr.265595.120)
Supplement: Supplemental Material [file supp_gr.265595.120_Supplemental_Fig_S21.pdf]

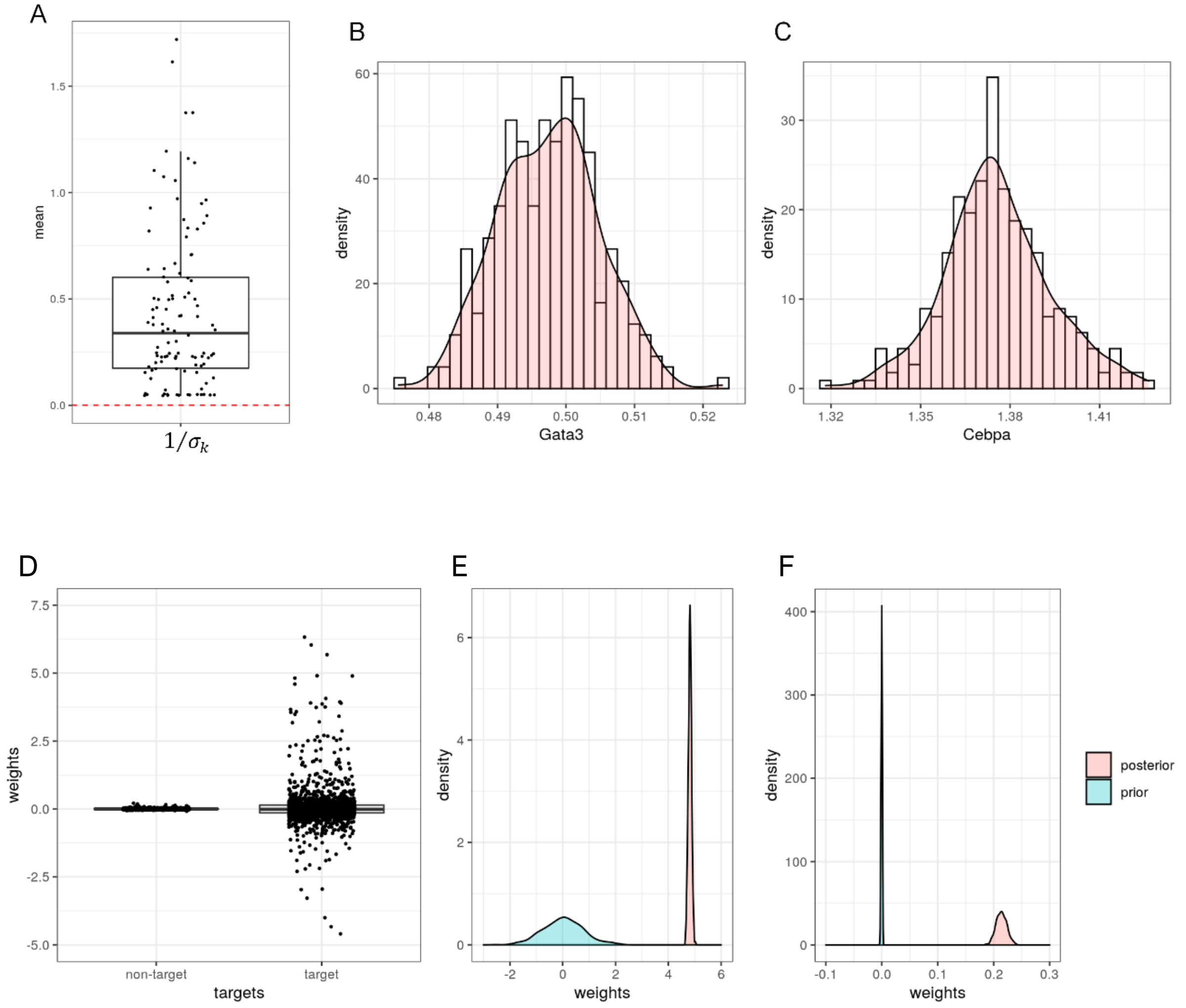

Figure. S21: The posterior distribution of $1/\sigma_k$ and weights:

A, Boxplot of posterior mean of $1/\sigma_k$ for every TF in the *Tabula Muris* lung dataset. **B**, The posterior distribution of $1/\sigma_k$ for GATA3-target pairs. **C**, The posterior distribution of $1/\sigma_k$ for CEBPA-target pairs. **D**, Boxplot of posterior mean of weights for TAL1 target gens in the *Tabula Muris* lung dataset. **E**, The prior and posterior distributions of weight between *Icam2* and TAL1. **F,** The prior and posterior distributions of weight between *Sdpr* and TAL1.

Fig.S21
